# The Hippo Pathway Regulates Homeostatic Growth of Stem Cell Niche Precursors in the *Drosophila* Ovary

**DOI:** 10.1371/journal.pgen.1004962

**Published:** 2015-02-02

**Authors:** Didem P. Sarikaya, Cassandra G. Extavour

**Affiliations:** Department of Organismic and Evolutionary Biology, Harvard University, Cambridge, Massachusetts, United States of America; The University of North Carolina at Chapel Hill, UNITED STATES

## Abstract

The Hippo pathway regulates organ size, stem cell proliferation and tumorigenesis in adult organs. Whether the Hippo pathway influences establishment of stem cell niche size to accommodate changes in organ size, however, has received little attention. Here, we ask whether Hippo signaling influences the number of stem cell niches that are established during development of the *Drosophila* larval ovary, and whether it interacts with the same or different effector signaling pathways in different cell types. We demonstrate that canonical Hippo signaling regulates autonomous proliferation of the soma, while a novel *hippo*-independent activity of Yorkie regulates autonomous proliferation of the germ line. Moreover, we demonstrate that Hippo signaling mediates non-autonomous proliferation signals between germ cells and somatic cells, and contributes to maintaining the correct proportion of these niche precursors. Finally, we show that the Hippo pathway interacts with different growth pathways in distinct somatic cell types, and interacts with EGFR and JAK/STAT pathways to regulate non-autonomous proliferation of germ cells. We thus provide evidence for novel roles of the Hippo pathway in establishing the precise balance of soma and germ line, the appropriate number of stem cell niches, and ultimately regulating adult female reproductive capacity.

## Author Summary

During development, organ growth must be carefully regulated to make sure that organs achieve the correct final size needed for organ function. In organs that are made of many different types of cells, this growth regulation is likely to be particularly complex, because it is important for organs to have appropriate proportions, or relative numbers, of the different kinds of cells that make up the organ, as well as the correct number of total cells. One method that cells use to regulate organ growth is a signaling pathway called the Hippo pathway. However, Hippo signaling has been studied, to date, primarily in organ systems that are made up of one cell type. In this study, we examine how Hippo signaling can work to regulate the proportions of different types of cells, as well as the total number of cells in an organ. To do this, we used the developing ovary of the fruit fly as a study system. We found that (1) Hippo signaling regulates the proliferation of many different cell types of the ovary; and (2) Hippo signaling activity in one cell type influences proliferation of other cell types, thus ensuring appropriate proportions of different ovarian cell types.

## Introduction

The Hippo pathway is a tissue-intrinsic regulator of organ size, and is also implicated in stem cell maintenance and cancer [1,2,3]. An outstanding question in the field is whether the Hippo pathway regulates proliferation of cells comprising stem cell niches during development in order to ensure that adult organs have an appropriate number of stem cells and stem cell niches [4]. The adult *Drosophila* ovary is an extensively studied stem cell niche system. In this organ, specialized somatic cells regulate the proliferation and differentiation of germ line stem cells (GSCs) throughout adult reproductive life [reviewed in 5]. The fact that GSCsare first established in larval stages raises the question of how the correct numbers of GSCs, and their associated somatic niche cells, are achieved during larval development. To date, only the Ecdysone, Insulin and EGFR pathways have been implicated in this process [6,7,8]. Here, we investigate the role of the Hippo pathway in regulating proliferation of somatic cells and GSC niche precursors to establish correct number of GSC niches.

Our current understanding of the Hippo pathway is focused on the core kinase cascade and upstream regulatory members. The Hippo pathway’s upstream regulation is mediated by a growth signal transducer complex comprising Kibra, Expanded and Merlin [9,10,11,12] and the planar cell polarity regulators Fat [13,14,15] and Crumbs [16,17]. Regulation of Hippo signaling further upstream of these factors appears to be cell type-specific [18]. When the core kinase cascade is active, the kinase Hippo (Hpo) phosphorylates the kinase Warts (Wts) [19,20]. Phosphorylated Wts then phosphorylates the transcriptional coactivator Yorkie (Yki), which sequesters Yki within the cytoplasm [21]. In the absence of Hpo kinase activity, unphosphorylated Yki can enter the nucleus and upregulate proliferation-inducing genes [21,22,23,24]. The Hippo pathway affects proliferation cell-autonomously in the eye and wing imaginal discs, glia, and adult ovarian follicle cells in *Drosophila* [18,19,20,25,26], as well as in liver, intestine, heart, brain, breast and ovarian cells in mammals [27,28,29,30,31,32]. Hippo pathway is often improperly regulated in cancers of these tissues, which display high levels and ectopic activation of the human ortholog of Yki, YAP [27,28,33,34]. Upregulation of YAP is also commonly observed in a variety of mammalian stem cell niches, where YAP can be regulated in a Hippo-independent way to regulate stem cell function [reviewed in 4]. Interestingly, germ line clones lacking Hippo pathway member function do not cause germ cell tumors in the adult *Drosophila* ovary, which has led to the hypothesis that Hippo signaling functions only in somatic cells but not in the germ line [35,36].

More recently, it has become clear that the Hippo pathway can regulate proliferation non-autonomously: Hippo signaling regulates secretion of JAK/STAT and EGFR ligands in *Drosophila* intestinal stem cells [37,38,39], and of EGFR ligands in breast cancer cell lines [31], and the resulting changes in ligand levels affect the proliferation of surrounding cells non-autonomously. How autonomous and non-autonomous effects of the Hippo pathway coordinate differentiation and proliferation of multiple cell types has nonetheless been poorly investigated. Moreover, most studies address the Hippo pathway’s role in adult stem cell function, but whether Hippo signaling also plays a role in the early establishment of stem cell niches during development remains unknown.

Here we use the *Drosophila* larval ovary as a model to address both of these issues. Adult ovaries comprise egg-producing structures called ovarioles, each of which houses a single GSC niche. The GSC niche is located at the anterior tip of each ovariole, and produces new oocytes throughout adult life. The niche cells include both GSC and differentiated somatic cells called cap cells [40]. Each GSC niche lies at the posterior end of a stack of seven or eight somatic cells termed terminal filaments (TFs). Somatic stem cells located close to the GSCs serve as a source of follicle cells that enclose each developing egg chamber during oogenesis [5]. All of these cell types originate during larval development, when the appropriate number of stem cells and their niches must be established. The larval ovary thus serves as a compelling model to address issues of homeostasis and stem cell niche development.

**Fig 1:**
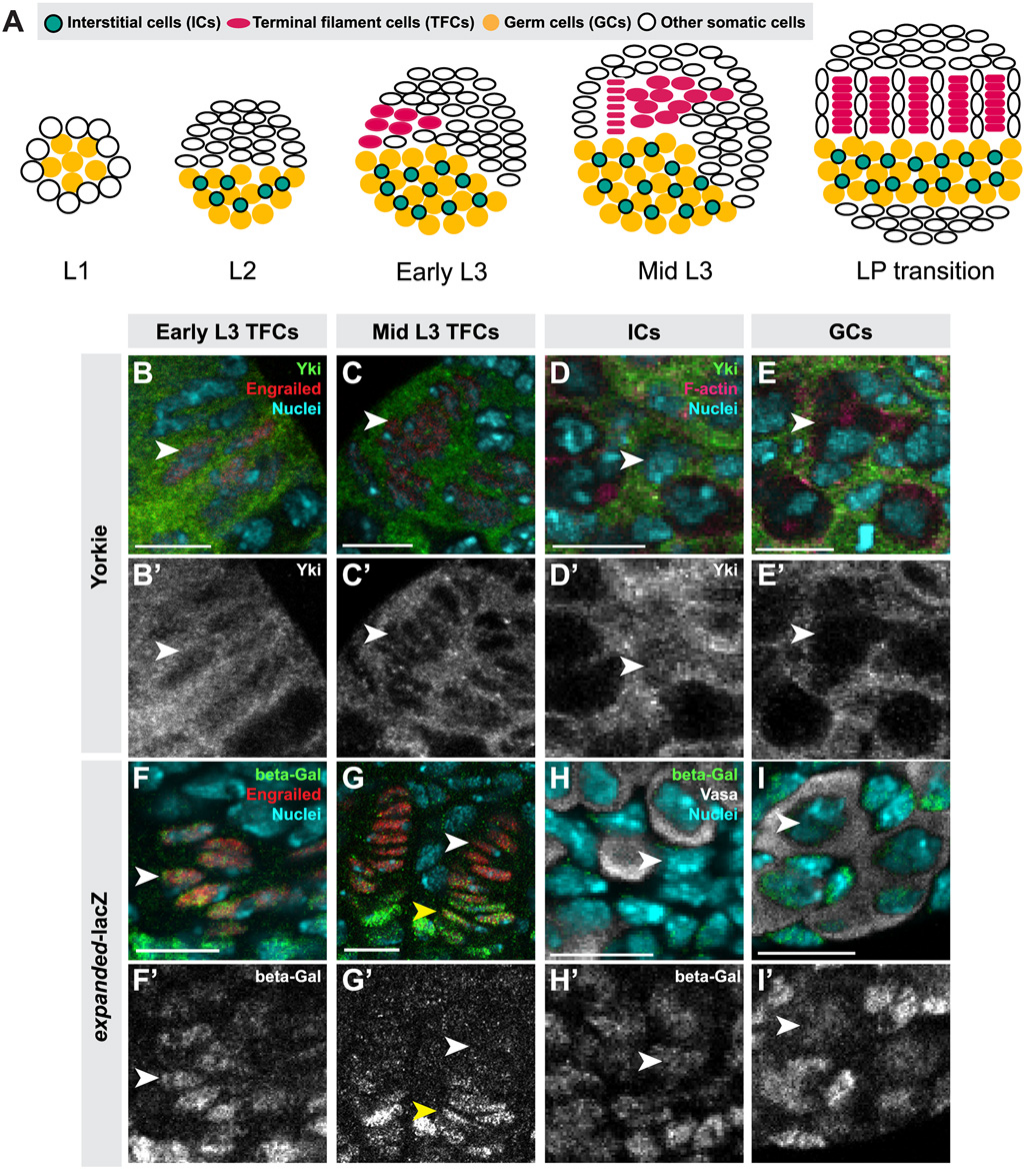
Hippo pathway activity is cell-type specific in the larval ovary. (A) Schematic of *Drosophila* larval ovarian development from first instar (L1) to the larval-pupal (LP) transition stage. The L1 larval ovary consists of germ cells (GCs: yellow) surrounded by a layer of somatic cells. As the ovary grows (L2), some somatic cells intermingle with GCs, becoming intermingled cells (ICs: green). Terminal filament cells (TFCs: pink) emerge during early L3, and begin intercalating to form terminal filaments (TFs), whose formation continues until the LP stage. (B–I, B’–I’) Expression of Yorkie (B–E) and *expanded*-*lacZ* (F–I) in larval ovarian cell types. B–I show merged images with Yki (B–E) or *ex-lacZ* (F–I) in green, nuclear marker Hoechst 33342 in cyan, TFC marker anti-Engrailed (B–C, F–G) or IC marker anti-Traffic Jam (D) in red, and F-actin (E) or GC marker anti-Vasa (H–I) in white. B’-I’ show Yki (B’-E’) or *ex-lacZ* (F’-I’) signal only. See S2I–J Fig. for quantification. (B) TFCs at early L3 that are intercalating into TFs display nuclear Yorkie localization. (C) Once incorporated into TFs, TFCs display cytoplasmic Yorkie localization. (D) ICs have high levels of nuclear and cytoplasmic Yorkie. (E) Yorkie is detectable only at very low levels in GCs. *expanded-lacZ* expression is detected in intercalating TFCs (F), ICs (H) and GCs (I) but not in TFCs once they are incorporated into TFs (G). White arrowheads indicate an example of the specific cell types indicated in each column. Yellow arrowheads indicate cap cells posterior to TFs. Scale bar = 10 μm in B–I’.

TFs serve as beginning points for ovariole formation and thus establish the number of GSC niches [41]. TFs form during third instar larval (L3) development by the intercalation of terminal filament cells (TFCs) into stacks (TFs) (Fig. 1A; [41]). TFCs proliferate prior to entering a TF, and cease proliferation once incorporated into a TF [42]. The morphogenesis and proliferation of TFCs during the third larval instar (L3) is regulated by Ecdysone and Insulin signaling, and by the BTB/POZ factor *bric-à-brac* (*bab*) [6,8,41,43,44,45]. Intermingled cells (ICs) arise from somatic cells that are in close contact with the germ cells (GCs) during L2, and proliferate throughout larval development [46] (Fig. 1A). ICs regulate GC proliferation and differentiation and are thought to give rise to escort cells in the adult niche [6,8,47]. Both Insulin and EGFR signaling promote the proliferation of ICs [6,48]. Finally, larval GCs give rise to GSCs and early differentiating oocytes. GCs proliferate during development and do not differentiate until mid-L3, when the GSCs are specified in niches that form posterior to the TFs [6,8,49], and the remaining GCs begin to differentiate as oocytes. GCs secrete Spitz, an EGFR ligand, and promote proliferation of ICs [49]. In addition, activation of Insulin and Ecdysone signaling in ICs regulates timing of early GC differentiation and cyst formation [6,8], though the identity of the IC-to-GC signal is unknown. ICs can non-autonomously regulate the proliferation of GCs both positively and negatively through Insulin and EGFR signaling respectively [6,7,49].

We previously showed that *hpo* and *wts* regulate TFC number in a cell-autonomous manner [50]. Here we demonstrate a role for canonical Hippo pathway activity in regulating both TFCs and ICs. We also provide evidence for three novel roles of Hippo pathway members in ovarian development: First, in contrast to a previous report suggesting that *yki* did not play a role in determining GC number [35], we show that non-canonical, *hpo*-independent *yki* activity regulates proliferation of the germ line. Second, we show that Hippo signaling regulates homeostatic growth of germ cells and somatic cells through the JAK/STAT and EGFR pathways. Third, we show that the Hippo pathway interacts with the JAK/STAT pathway to regulate TFC number, and with both the EGFR and JAK/STAT pathways to regulate IC number autonomously and GC number non-autonomously. These data elucidate how Hippo pathway-mediated control of ovarian development establishes an organ-appropriate number of stem cell niches, and thus ultimately influences adult reproductive capacity.

## Results

### Hippo pathway activity is cell type-specific in the larval ovary

To determine whether Hippo signaling regulates proliferation of the GSC niche precursor cells, we first examined the expression pattern of Hippo pathway members in the larval ovary. Throughout larval development, Hpo was expressed ubiquitously in the ovary (S1A–C Fig.), and Yki was expressed in all somatic cells of the ovary (S1F–H Fig.). However, the subcellular localization of Yki was dynamic during ovariole morphogenesis, and different in distinct somatic cell types. We observed nuclear Yki expression in newly differentiating TFCs (identified by Engrailed expression and elongated cellular morphology) (Fig. 1B–B’, arrowhead; S2I Fig.), while late stage TFs had very little detectable nuclear Yki (Fig. 1C–C’, arrowhead; S2I Fig.). Since Yki localization in the nucleus indicates low or absent Hippo pathway activity [21], these data suggest that Hpo signaling may promote TFC and TFC-progenitor proliferation before TF formation, and then suppress proliferation in TFCs that have entered TFs. This is consistent with previous reports of the somatic proliferative dynamics of the larval ovary [42,48].

We also assessed Yki activity by analyzing expression of the downstream target genes *expanded* (*ex*) [21], *diap1* (also called *thread*) [21] and *bantam* [22]. *ex-lacZ* (Fig. 1F–G, S2J Fig.) and *diap1-lacZ* (S2A–B, K Fig.) were expressed in early TFCs, but ceased expression once TFCs were incorporated into a TF. The *bantam*-*GFP* sensor is a GFP construct containing *bantam* miRNA target sites, such that low or absent GFP expression indicates *bantam* expression and activity. The sensor was not expressed in early differentiating TFCs, but was expressed in TFCs within a TF (S2E–F, L Fig.). These data are consistent with the subcellular localization of Yki in TFCs. Yki activity reporters were also expressed in cap cells of the GSC niche, which are immediately posterior to TFs (Fig. 1G–G’, yellow arrowhead).

In ICs, strong cytoplasmic and nuclear expression of Yki was observed throughout development (Fig. 1D–D’; S2I Fig.). Likewise, all Yki activity reporters examined were expressed in ICs (Fig. 1H–H’, S2C–C’, G–G’, J–L Figs.), consistent with continuous proliferation of these cells throughout larval development.

### Hippo signaling regulates terminal filament cell proliferation

The expression patterns described above, and our previous observation that knockdown of *hpo* or *wts* increased TFC number [50], suggested that the Hippo pathway regulates TFC proliferation. To further test this hypothesis, we manipulated activity of the core Hippo pathway members *hpo*, *wts* and *yki* in somatic cells using the *bric-à-brac* (*bab)* and *traffic jam (tj) GAL4* drivers [51,52]. *bab*:*GAL4* is strongly expressed in TFCs during L3 but only weakly in other somatic cell types [50,51]. *tj*:*GAL4* is expressed primarily in somatic cells posterior to the TFs, including ICs, to a lesser extent in newly forming TF stacks during early and mid L3, and in posterior TFCs in late L3 (S3A–D Fig.) [53]. We note that the expression of Tj in intercalating TFCs is not detected with the Traffic-Jam antibody (S3E Fig.). Antibody staining against Hpo and Yki was used to confirm effectiveness of the RNAi-mediated knockdown under both GAL4 drivers (S1D–D’, I–I’ Fig.; see Methods for further details of RNAi validation in these and subsequent experiments).

**Fig 2:**
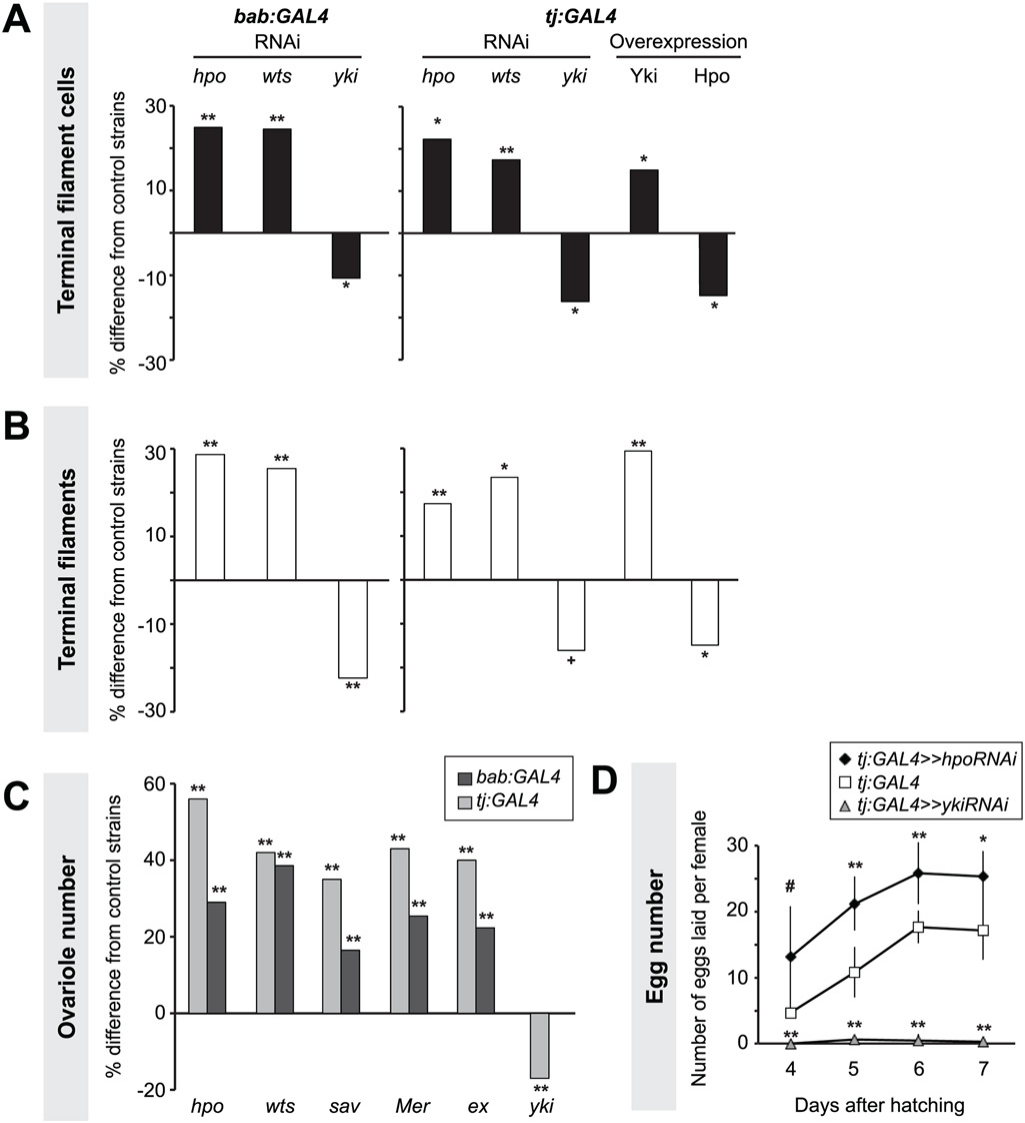
Hippo pathway influences proliferation of TFCs, thereby influencing ovariole number. Changes in (A) TFC or (B) TF number in LP ovaries expressing UAS-induced RNAi against *hpo*, *wts* or *yki*, or overexpressing *hpo* or *yki* under the *bab*:*GAL4* or *tj*:*GAL4* drivers. Here and in Figs. 3–6, S4 and S5, bar graphs show percent difference from control genotypes of the indicated cell type or structure in each of the experimental genotypes, which are those that carry both *UAS* and *GAL4* constructs. Control genotypes are either parental strains or siblings carrying a balancer chromosome instead of the GAL4 construct (see Methods). When statistical comparisons were performed to parental strains, values from the two parental strains were averaged and percent difference from the average was plotted. Statistical significance was calculated using a student’s two-tailed t-test with unequal variance. ** *p*<0.01, * *p*<0.05, + *p*<0.01 against the UAS parental line and *p* = 0.08 against the GAL4 parental line. n = 10 for each genotype. Numerical data can be found in S1 Table. (C) Changes in adult ovariole number in individuals expressing *hpo*, *wts*, *sav*, *Mer*, *ex* or *yki* RNAi under *bab*:*GAL4* (dark grey bars) or *tj*:*GAL4* (light grey bars) drivers. ** *p*< 0.01 against controls. n = 20 for each genotype. (D) Average egg counts from females four to seven days after hatching of *tj*:*GAL4* (control, white squares), *tj*:*GAL4* driving *hpo* ^*RNAi*^ (black diamonds), *tj*:*GAL4* driving *yki* ^*RNAi*^ (grey triangles). Error bars indicate confidence intervals. * *p*<0.05, ** *p*<0.01. n = 5 vials containing 3 females per vial.

Lowering Hippo pathway activity in somatic cells by expressing RNAi against *hpo* or *wts* under either GAL4 driver significantly increased TFC number (student’s t-test was used for this and all other comparisons: *p*<0.05; Fig. 2A, S1 Table). We previously showed that TFC number correlates with TF number [50]. Accordingly, driving RNAi against either *hpo* or *wts* in somatic tissues significantly increased TF number (*p*<0.05; Fig. 2B, S1 Table). Conversely, decreasing Yki activity in somatic cells by expressing *yki* RNAi under either driver significantly reduced both TFC number and TF number (*p*<0.05; Fig. 2A–B, S1 Table).

Somatic overexpression of *hpo* or *yki* under the *bab*:*GAL4* driver resulted in larval lethality, likely due to the known pleiotropic expression of *bab* in multiple non-ovarian tissues [51]. However, *tj*:*GAL4*-driven overexpression of *yki* or *hpo* was viable. Using the *tj*:*GAL4* driver, we found that somatic *yki* overexpression resulted in a significant increase in both TFC number (*p*<0.05; Fig. 2A, S1 Table) and TF number (*p*<0.01; Fig. 2B, S1 Table), while somatic *hpo* overexpression led to a significant reduction in both TFCs and TFs (*p*<0.05; Fig. 2A–B, S1 Table). A null allele of the Hippo pathway effector *expanded* (*ex* ^*1*^ [54]) and a gain of function allele of *yorkie* (*yki* ^*DB02*^ [33]) both led to a significant increase in TFC number (S4A Fig., S2 Table), consistent with results obtained from RNAi treatments.

As larval TF number corresponds to the number of GSC niches in the adult (ovariole number) [50], we asked if Hpo signaling might play a role in determining ovariole number. We quantified ovariole number in adults with RNAi-mediated knockdown of Hippo signaling pathway members *hpo*, *wts*, *salvador* (*sav*), *Merlin* (*Mer*), or *ex* in somatic cells. In all cases adult ovariole number was significantly increased (*p*<0.01; Fig. 2C). Conversely, *yki* knockdown under *tj*:*GAL4* significantly decreased ovariole number (*p*<0.01; Fig. 2C).

Adult females of all reported somatic knockdown and overexpression experiments were viable and did not have defects in adult ovarian structure. Adult females expressing *hpo* RNAi under the *tj*:*GAL4* driver, which had significantly more ovarioles than controls (Fig. 2B, S1 Table), also laid significantly more eggs than controls (Fig. 2D). Conversely, adult females expressing *yki* RNAi under *tj*:*GAL4* driver laid significantly fewer eggs than controls, and some appeared to be entirely sterile (Fig. 2D). This shows that by regulating somatic gonad cell number in the larval ovary, the Hippo pathway can influence adult female reproductive capacity.

### Hippo signaling regulates intermingled cell proliferation

**Fig 3:**
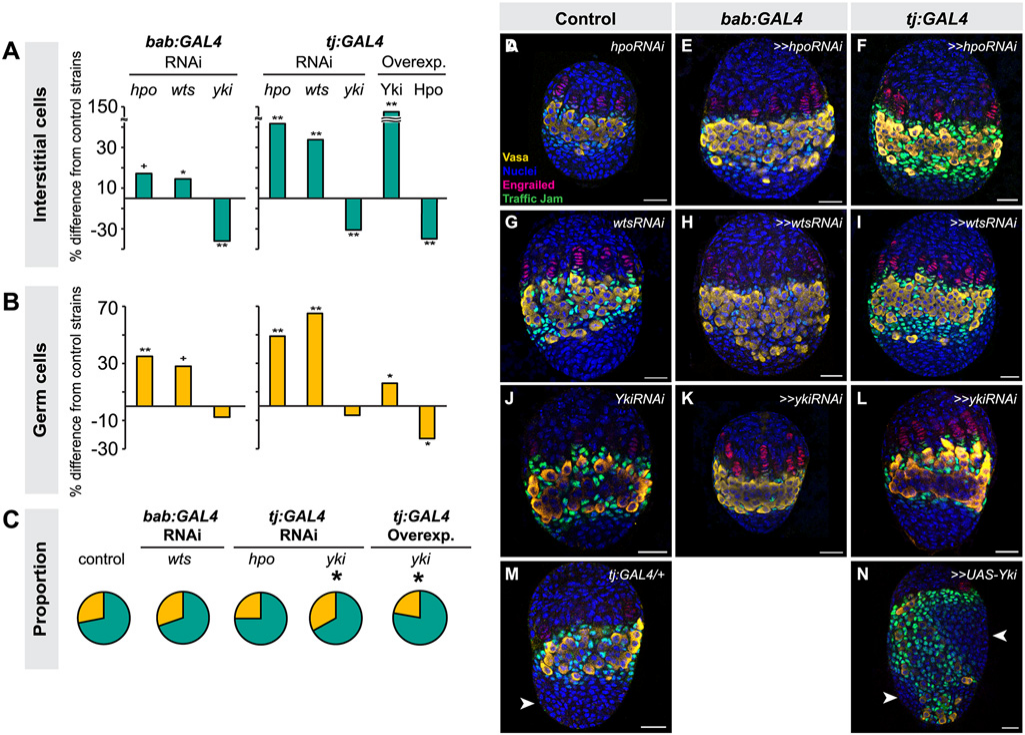
Altering Hippo pathway activity in somatic cells changes IC and GC number. Changes in (A) IC or (B) GC number in ovaries expressing *hpo*, *wts* or *yki* RNAi, or overexpressing *hpo* or *yki* under the *bab*:*GAL4* or *tj*:*GAL4* drivers. Bar graphs are as explained in Fig. 2 legend. ** *p*<0.01, * *p*<0.05, + *p* = 0.05 against the UAS parental line and *p*<0.05 against the GAL4 parental line. n = 10 for each genotype. Numerical values can be found in S3 Table. (C) Pie charts of proportions of ICs (green) and GCs (yellow) in ovaries under indicated selected experimental conditions. * *p*<0.05. Numerical values can be found in S6 Table; pie charts for all experimental conditions shown in S7 Fig. (D–N) LP stage larval ovaries representative of control and experimental samples used to obtain cell type counts. Scale bar = 10 μm.

We next asked whether the Hippo pathway also influenced the proliferation of ICs, which do not contribute to TF formation but are in direct contact with germ cells and are thought to give rise to somatic stem cells or escort cells [6,8,47]. Larval-pupal transition (LP) stage ICs were identified by antibody staining against Traffic Jam, which is specific to ICs at this stage of development (Lin et al., 2003). Altering Hippo pathway activity in somatic cells had the same overall effects on IC number as on TFC number: knocking down *hpo* or *wts* or overexpressing Yki resulted in a significant increase in IC number (*hpo* or *wts* RNAi: *p*<0.05 for *bab*:*GAL4* and *p*<0.01 for *tj*:*GAL4*; *yki* overexpression: *p*<0.01 for both drivers; Fig. 3A, D–I, M–N S3 Table). Conversely, RNAi against *yki* or overexpression of *hpo* in the soma significantly reduced IC number (*p*<0.01; Fig. 3A, J–L, S3 Table). As observed for TFC number, IC numbers in *ex* ^*1*^ or *yki* ^*DB02*^ backgrounds were significantly increased (S4 Fig., S2 Table), consistent with the RNAi data. Ovarian morphogenesis, including TF, ovariole and GSC niche formation, was normal in most cases (Fig. 3D–N). However, the 150% increase in IC number caused by *yki* overexpression correlated with failure of swarm cell migration in some ovaries (Fig. 3M–N, arrowhead; n = 2/10), suggesting that excessive proliferation of ICs above a certain threshold cannot be accommodated by the ovary, leading to disrupted ovariole morphogenesis.

Because the *tj*:*GAL4* and *bab*:*GAL4* drivers are expressed in both ICs and TFCs (albeit at varying levels), we could not use these tools to determine whether ICs and TFCs influence each other’s proliferation non-autonomously. Thus, we tested the utility of *ptc*:*GAL4*, which is expressed in ICs (albeit at low levels) but not in TFCs (S5A–C Fig.), and *hh*:*GAL4*, which is expressed in a subset of TFCs during and after TF stacking but not in ICs (S5D–F Fig.), for this purpose. However, when we drove RNAi against *hpo* or *wts* using either driver, we did not observe any significant changes in IC, TFC or TF number compared to controls (S5G–H Fig.; S5 Table). This is likely due to the facts that (1) *hh*:*GAL4* expression in TFCs arises after TFC proliferation has essentially completed (S5D–F Fig.); and (2) *ptc*:*GAL4* expression is extremely weak in ICs (S5A–C Fig.). We therefore cannot rule out the hypothesis that TFC or IC proliferation has a non-autonomous influence on the other of these two somatic cell types.

### Germ cell proliferation is regulated by *yki* in a *hpo/wts*-independent manner

Having observed apparently canonical Hippo pathway activity in the somatic gonad cells, we next asked whether this pathway operated similarly in germ cells, and found a number of significant differences. First, unlike the dynamic expression of Yki in somatic ovarian cells, we detected only extremely low levels of Yki in GCs throughout development (Figs. 1E, E’, S1F–H, S2I). The *bantam*-GFP sensor also suggested low or absent Yki activity in GCs (S2H–H’, L Fig.). However, we did observe expression of the *expanded*-*lacZ* (Figs. 1I–I’, S2J) and *diap1*-*lacZ* (S2D–D’, S2K Figs.) reporters in the GCs. We thus performed functional experiments to evaluate the roles of Yki and other Hpo pathway members in GCs.

**Fig 4:**
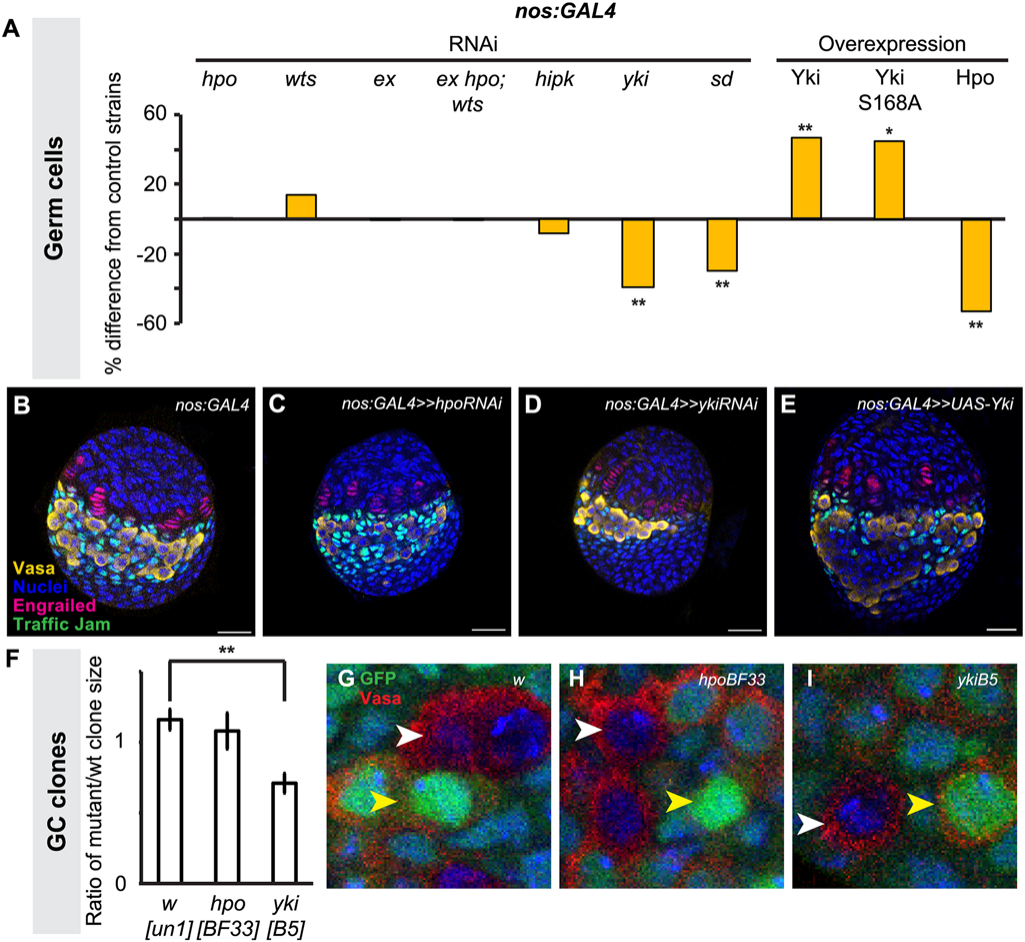
Yorkie activity regulates GC number. Changes in (A) GC number in ovaries expressing *hpo*, *wts*, *ex*, *hpo/wts/ex* triple, *hipk*, *yki or sd* RNAi, or overexpressing *hpo*, *yki or yki* ^*S168A*^ under the *nos*:*GAL4* driver. Bar graphs are as explained in Fig. 2 legend. * *p*<0.05, ** *p*<0.01 against controls. n = 10 for each genotype. Numerical values can be found in S2 Table. (B–E) LP stage larval ovaries representative of control and experimental samples used to obtain cell type counts. Scale bar = 10 μm. (F) Ratio of size (number of cells per clone) of homozygous mutant versus homozygous wild type twin spot clones for control (*w* ^*un-1*^), *hpo* ^*BF33*^ and *yki* ^*B5*^ alleles. ** *p*<0.01 against control. (G–I) LP stage larval ovaries representative of control and experimental samples for clonal analysis showing GCs (Vasa, red), homozygous wild type clones (strong GFP expression; yellow arrowhead), and clones homozygous for tested alleles (no GFP; white arrowhead). n = 10 for each genotype.

We disrupted Hippo pathway activity in GCs using the germ line-specific driver *nos*:*GAL4* (S3E–H Fig.). In contrast to the overproliferation of somatic cell types observed in the experiments described above, driving RNAi against *hpo* or *wts* in the germ line did not significantly change GC number (Fig. 4A; S3 Table). However, driving *yki* RNAi in the germ line significantly reduced GC number (*p*<0.01; Fig. 4A), and a second independent RNAi line [55,56] yielded similar results (p<0.05; S3 Table). Conversely, overexpression of *yki* in GCs led to a significant increase in GCs (*p*<0.01, Fig. 4A). Although *hpo* RNAi had no effect on GC number (Fig. 4A, S3 Table), *hpo* overexpression significantly decreased GC number (*p*<0.01; Fig. 4A). Interestingly, we observed a non-autonomous increase in ICs in when *yki* was overexpressed in GCs, but not in the other experimental conditions (*p*<0.05, S3 Table).

To validate our findings from the *hpo* and *yki* RNAi experiments, we induced *hpo* [57] and *yki* [21] null mutant GC clones in L1 larvae and compared the clone sizes (number of cells per clone) of homozygous mutant clones and their homozygous wild type twin spot clones in late L3 ovaries. Consistent with our RNAi analysis, *hpo* ^*BF33*^ clones were not significantly different in size from controls (Fig. 4F, H), but *yki* ^*B5*^ clones were significantly smaller than controls (*p*<0.01; Fig. 4F, I). Taken together, both RNAi and clonal analysis data suggest that *yki* but not *hpo* is involved in regulating GC number.

We therefore sought further evidence that *yki* activity in the germ line was independent of *hpo*. The FERM domain protein Expanded can bind to Yki independently of Hpo or Wts to sequester Yki to the cytoplasm of *Drosophila* eye imaginal disc and S2 cells [58], or alternatively can bind to and sequester Yki by forming a complex with Hpo and Wts in *Drosophila* wing imaginal discs [59]. To determine if one of these mechanisms might be operating in GCs, we knocked down *ex* alone, or *hpo*, *wts* and *ex* together in GCs. We did not observe significant changes in GC number under either condition (Fig. 4A; S3 Table), suggesting that these phosphorylation-independent mechanisms do not regulate Yki in GCs. Consistent with this hypothesis, we found that overexpression of *yki* ^*S168A*^, an allele of Yki that is impervious to Wts-mediated phosphorylation [60], also significantly increased GC number (Fig. 4A). To our knowledge, the only other identified *hpo*-independent mechanism of *yki* regulation in *Drosophila* is via the kinase Hipk, which phosphorylates Yki and induces nuclear translocation in *Drosophila* wing imaginal discs [61]. However, knocking down *hipk* in GCs also did not affect GC number (Fig. 4A). Finally, we asked if Yki might still operate together with the transcription factor Scalloped (Sd)/TEAD in germ cells, as has been shown in somatic cells of *Drosophila* and mammals [24,62,63]. Knocking down *sd* in GCs significantly reduced GC number (*p*<0.01, Fig. 4A), suggesting that a Yki/Sd complex could play a role in GC proliferation.

A reduction in GC number could be caused by altered GC proliferation, or by premature differentiation of GCs into oocytes, as has been observed for loss of function mutations in members of the Ecdysone and Insulin signaling pathways [6,8]. To ask if altered *yki* activity was causing changes in GC number by affecting the timing of oocyte differentiation, we assayed for fusome morphology, an indicator for early cyst cells, in ovaries expressing RNAi against *yki* or overexpressing *yki* in GCs (S6 Fig.). We observed no overt signs of early differentiation of PGCs and fusome morphology was similar to controls, suggesting that the reduction of GC number induced by *yki* knockdown in GCs is likely due to reduced GC proliferation.

### Changing Hippo pathway activity in the soma non-autonomously influences GC number

Given our finding that Hippo signaling pathway members regulate autonomous proliferation of both somatic and germ line cells, we asked if this pathway might also coordinate non-autonomous proliferation of both cell types. Such a mechanism might be expected to operate in order to adjust the numbers of one cell type in response to Hippo signaling-mediated changes in the other, which would ensure an appropriate number of operative stem cell niches [8]. To test this hypothesis, we analyzed GC number in conditions where Hippo pathway activity was altered in the somatic cells. Non-autonomous positive regulation of GC number by ICs has been documented, but only in ways that also affect GC differentiation [49]. Whether ICs can positively regulate GC proliferation without affecting their differentiation thus remains unknown [6,8]. We found that increasing somatic cell number by driving *hpo* or *wts* RNAi in the soma also significantly increased GC number (*p*<0.01, *p* = 0.06 respectively; Fig. 3B–I; S3 Table). Strikingly, GC number increased in precise proportion to the IC number increase, whether this increase was as little as 15% (*bab*:*GAL4>>wts* ^*RNAi*^; Figs. 3C, S7; S3, S6 Tables) or as much as 70% (*tj*:*GAL4>>hpo* ^*RNAi*^; Figs. 3C, S7; S3, S6 Tables), resulting in a consistent ratio of ICs to GCs (Figs. 3C, S7; S6 Table). However, increasing IC number by 150% via somatic overexpression of *yki* prompted only a 10% increase in GC number (*p*<0.05, Fig. 3B). In this condition, the GC:IC ratio was significantly lower than controls (Figs. 3C, S7; S6 Table), and GC:IC proportions were not maintained (Figs. 3C, S7). These results suggest that the Hippo pathway can maintain homeostatic growth of the larval ovary by regulating the number of GCs to accommodate changes of up to 70% in the number of ICs. However, further overproliferation of ICs cannot be matched by proportional GC proliferation.

We then asked if somatic Hippo signaling could also non-autonomously compensate for decreases in IC number via a proportional reduction in GC number. We found that somatic *yki* RNAi significantly decreased IC number (*p*<0.05), but did not significantly decrease GC number (*p* = 0.29, Fig. 3B), thus disrupting the GC:IC ratio (Figs. 3C, S7). However, reducing IC number via *hpo* overexpression in the soma yielded a marginally significant decrease in GC number (Fig. 3A, B). These results suggest that the Hippo pathway’s role in non-autonomous proliferation of GCs is primarily operative in cases of somatic cell overproliferation, but that to accommodate significant decreases in IC number by reducing GC numbers, Hippo signaling is not always sufficient and additional mechanisms may be required. The latter may include insulin signaling [6].

### *hpo* interacts with EGFR signaling in ICs but not TFCs

**Fig 5:**
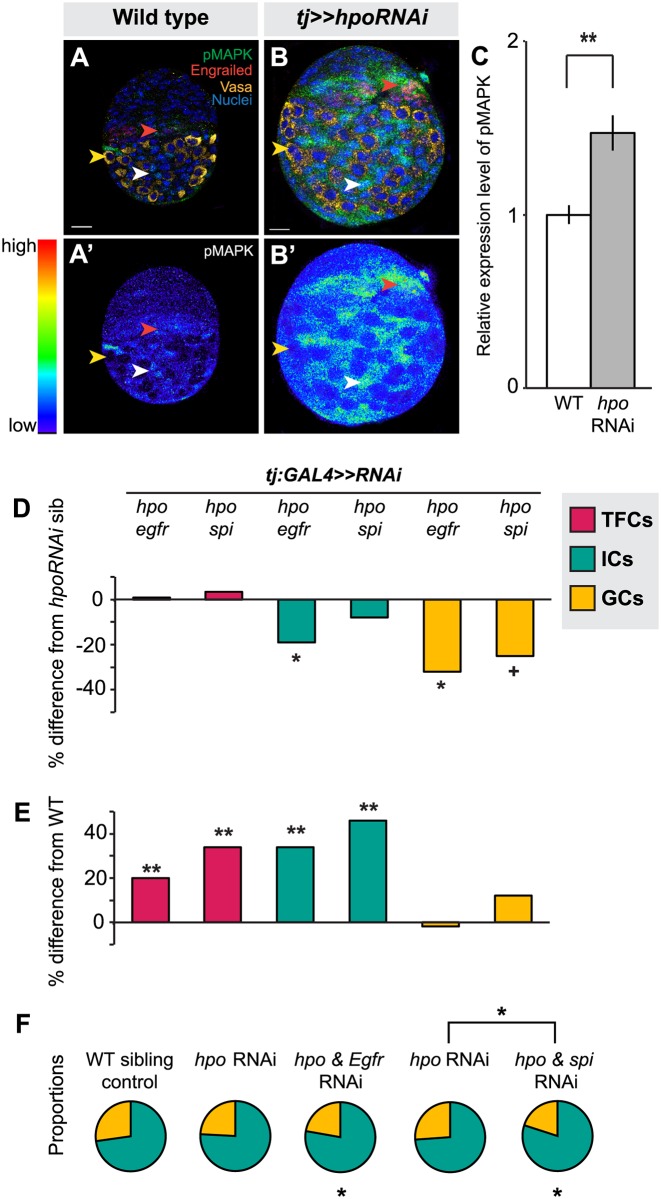
The Hippo pathway interacts with the EGFR pathway to regulate IC and GC growth. (A) Expression pattern of EGFR pathway activity marker pMAPK in wild type L3 ovary. Expression is mainly in posterior IC cells. Scale bar = 10 μm and applies also to A’. (B) pMAPK expression in ovary expressing *UAS*:*hpo* ^*RNAi*^ in the soma, exposed at same laser setting as (A). Scale bar = 10 μm and applies also to B’. (C) Relative intensity of anti-pMAPK fluorescence in wild type compared to *hpo* knockdown experimental (n = 8). Overall expression level of pMAPK is higher than controls, most prominently in the ICs. (D–E) Percent difference in TF (red), IC (green), and GC (yellow) number in double RNAi (*hpo* and *egfr*, or *hpo* and *spi*) compared to *hpo* single RNAi sibling controls (D), and wild type sibling controls (E). * *p*<0.05, ** *p*<0.01. Numerical values can be found in S4 Table. (F) Pie charts showing proportions of ICs (green) and GCs (yellow) under indicated selected experimental conditions. * *p*<0.05, see S6 Table for numerical values; pie charts for all experimental conditions shown in S7 Fig.

Finally, we asked which signaling pathways Hippo signaling might interact with in the ovary to regulate proliferation. We also asked whether these pathways were the same or different in distinct somatic cell types (ICs and TFCs). First, we considered the EGFR pathway. The Hippo pathway interacts with the EGFR pathway to regulate non-autonomous control of proliferation in other organs [31,38,64,65,66]. Moreover, the EGFR pathway is known to regulate IC number and to non-autonomously regulate GC number [7,49]. We therefore asked whether the Hippo pathway interacted with EGFR signaling in the larval ovary. In wild type larval ovaries we observed, as previously reported [49], that pMAPK (a readout of EGFR activity) is expressed predominantly in ICs (Fig. 5A, white arrowhead) and in some TFCs (Fig. 5A, red arrowhead), but not in GCs (Fig. 5A, yellow arrowhead). When we knocked down *hpo* in the soma, we detected significantly increased pMAPK expression in the ovary (*p*<0.01; Fig. 5B–C), most notably in ICs at mid L3 and late L3 stages (Fig. 5B, white arrowhead), and additionally in some TFCs (Fig. 5B, red arrowhead). These results suggest that in wild type ovaries Hippo pathway activity may limit EGFR activity in somatic cells.

In order to assess the consequences of *hpo*/EGFR pathway interactions, we conducted double-RNAi knockdowns of *hpo* and either the EGFR receptor (*egfr*) or the EGFR ligand *spitz* (*spi*) in the soma using the *tj*:*GAL4* driver. To validate the RNAi constructs, we expressed *egfr* ^*RNAi*^ or *spi* ^*RNAi*^ under *tj*:*GAL4*, and observed significant reduction in pMAPK levels in L3 ovaries (p<0.05, S8A Fig.). In both *hpo* and *egfr* or *spi* double-RNAi knockdowns, TFC number was not significantly different from *hpo* single knockdowns (Fig. 5D; S4 Table). In addition, TFC number was not altered when we knocked down *egfr* or *spi* alone in the soma (S4 Table). This suggests that the Hippo pathway does not regulate TFC number via EGFR signaling, consistent with the limited pMAPK expression observed in TFCs (Fig. 5A, red arrowhead).

In contrast, and consistent with the strong pMAPK expression in ICs (Fig. 5A, white arrowhead), *tj*:*GAL4-*mediated double knockdown of *hpo* and *egfr* partially rescued the *hpo* RNAi-induced overgrowth of ICs (*p*<0.05; Fig. 5D; S4 Table). However, these ovaries still had 35% more ICs than wild type controls (*p*<0.01; Fig. 5E). Double knockdown of *hpo* and *spi* yielded no significant difference in IC number compared to *hpo* single knockdowns (Fig. 5D). IC number was unaltered by knockdown of *egfr* or *spi* alone (S4 Table). In contrast to the TFCs, the Hippo pathway thus appears to interact with EGFR signaling to regulate IC number.

Finally, we quantified GCs to test whether the EGFR-Hippo signaling interaction in ICs could non-autonomously regulate GCs. Double knockdown of *hpo* and *egfr*, which significantly reduced IC number relative to *hpo* RNAi alone (*p*<0.05; Fig. 5D; S4 Table), also resulted in significantly fewer GCs (*p*<0.05; Fig. 5D; S4 Table), completely rescuing the *hpo* RNAi-induced GC overproliferation (Fig. 5E; S4 Table). Double knockdown of *hpo* and *spi* did not alter IC number relative to *hpo* RNAi alone (*p* = 0.24; Fig. 5D; S4 Table), but resulted in near-significant reduction of GCs (*p* = 0.054), also yielding a complete rescue of the *hpo* RNAi-induced overproliferation (Fig. 5E; S4 Table). Because the degree of *hpo* RNAi rescue was greater in GCs than in ICs in the *hpo*/*spi* double knockdown, the homeostatic balance of these cell types was no longer maintained (S5F Fig.). As previously reported [7,49], we observed a significant increase in GC number when we knocked down *egfr* alone, but not *spi* alone, in the soma (S4 Table). Taken together, these results indicate that *hpo* interacts in the soma with *egfr* signaling, likely through an additional ligand along with *spi*, to regulate both IC number autonomously and GC number non-autonomously.

### *hpo* interacts with JAK-STAT signaling in TFCs and ICs

**Fig 6:**
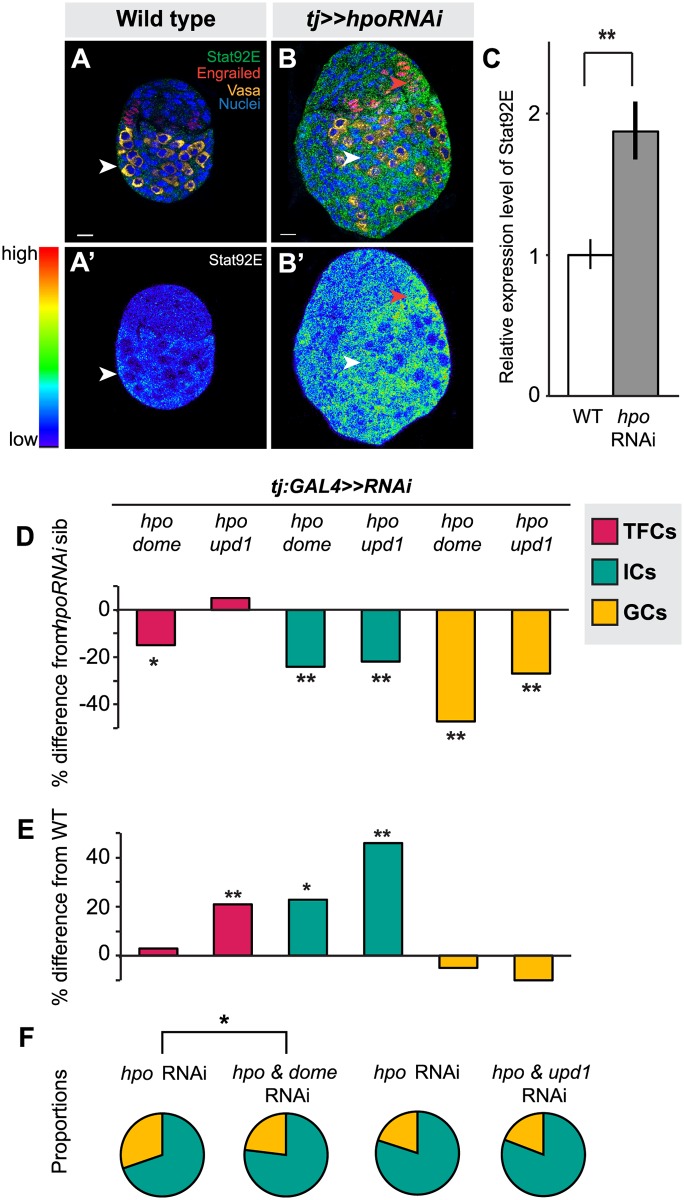
Hippo pathway interacts with JAK/STAT pathway to regulate TFC and IC proliferation, and non-autonomous regulation of GC number. (A) Expression pattern of JAK/STAT pathway kinase Stat92E in wild type L3 ovary. Scale bar = 10 μm and applies also to A’. (B) Stat92E expression in ovary expressing *UAS*:*hpo* ^*RNAi*^ in the soma, exposed at same laser setting as (A). Scale bar = 10 μm and applies also to B’. (C) Relative intensity of anti-Stat92E fluorescence in wild type compared to *hpo* knockdown experiments (n = 10). (D–E) Percent difference in TF (red), IC (green), GC (yellow) number in double RNAi (*hpo* and *dome*, or *hpo* and *upd1*) knockdowns compared to *hpo* single RNAi sibling controls (D), and wild type sibling controls (E). * *p*<0.05, ** *p*<0.01. Numerical values can be found in S4 Table. (F) Pie charts showing proportions of ICs (green) and GCs (yellow) of *hpo* RNAi control and *hpo* and *dome/up1* double RNAi. * *p*<0.05, see S6 Table for numerical values; pie charts for all experimental conditions shown in S7 Fig.

Another characterized interacting partner of the Hippo pathway in various somatic tissues is the JAK/STAT pathway [37,38,39,67,68,69]. We therefore asked whether these two pathways also interact to regulate autonomous and/or homeostatic proliferation in the larval ovary. First, we used detection of Stat92E as a readout of JAK/STAT activity [70,71,72]. We observed strongest Stat92E expression in posterior somatic cells, including ICs, in wild type ovaries (Fig. 6A–A’). Knocking down *hpo* in the soma led to significantly higher Stat92E levels (*p*<0.01; Fig. 6B–C), suggesting that, similar to its interaction with EGFR, Hippo pathway activity normally limits JAK/STAT pathway activity in the larval ovary.

Next, we asked if RNAi against either the JAK/STAT receptor *dome* or the ligand *unpaired* (*upd1*) could rescue the effects of *hpo* RNAi in the soma. While there are three *upd* orthologues in *Drosophila* [73,74,75], we focused on *upd1*, as it is known to regulate GC proliferation in the testis [76] and thought to be a specific *yki* target in polar cells [77], which are derivatives of the somatic cells of the ovary. Expressing *dome* or *upd1* RNAi under *tj*:*GAL4* significantly reduced Stat92E levels in the larval ovary (*p*<0.05 for *dome*, *p* = 0.06 for *upd1*; S8B Fig.), confirming functionality of these RNAi lines. In TFCs, double knockdown of *dome* and *hpo*, but not of *upd1* and *hpo*, completely suppressed the *hpo* single knockdown phenotype (*p*<0.05; Fig. 6D; S4 Table). Knocking down *dome* alone in the soma significantly decreased TFC number (*p*<0.05; S4 Table), supporting the hypothesis that JAK/STAT signals positively regulate TFC proliferation. These data suggest that Hippo signaling regulates TFC proliferation via interactions with the JAK/STAT pathway, and that a ligand other than *upd1* mediates this interaction.

We next counted IC and GC number to determine whether JAK/STAT-Hippo pathway interactions regulate ICs proliferation autonomously, and/or GC proliferation non-autonomously. Both *dome*/*hpo* or *upd1*/*hpo* double knockdowns partially rescued the overproliferation caused by knockdown of *hpo* alone (*p*<0.01; Fig. 6D; S4 Table), but these ovaries still had significantly more ICs than wild type controls (*p*<0.05; Fig. 6E). Both double knockdown conditions also completely rescued the non-autonomous increase in GC number caused by *hpo* RNAi (Fig. 6D; S4 Table). Similar to our experiments on the EGFR pathway, we observed abnormal IC:GC ratios in the *hpo* and *dome* RNAi single knockdowns (Figs. 6F, S7; S6 Table). In summary, Hippo signaling interacts with JAK/STAT signaling via *upd1* to regulate IC:GC homeostasis.

## Discussion

### Hippo signaling in somatic cells of the larval ovary

We have shown that canonical cell autonomous Hippo signaling regulates proliferation of two key somatic cell types, TFCs and ICs. Because TFCs form TFs, which are the beginning points of each GSC niche, the number and stacking of TFCs can ultimately influence adult ovariole number and thus reproductive capacity [50,78,79]. We previously showed that the differences in ovariole number between *D*. *melanogaster* and closely related *Drosophila* species results from changes in TFC number [48,50]. This suggests Hippo and JAK/STAT pathway members as novel potential targets of evolutionary change in ovariole number variation. Indeed, loci containing many of these genes have been previously identified in QTL analyses of genomic variation correlated with ovariole number variation [80]. Our study thus provides novel experimental validation of previous quantitative genetics approaches to understanding the genetic regulation of ovariole number.

In TFCs, Hippo signaling regulates proliferation by interacting with *dome* but not *upd1*, suggesting that one or both of *upd2* or *upd3* act as ligands for JAK/STAT signaling in this context. Alternatively, a role for *upd1* in TFC number regulation may have been obscured by our use of the *tj*:*GAL4* driver, since this driver is restricted to cells posterior to TFCs in L3. A potential source of JAK/STAT ligands that would not have been captured by our experiments could be the anterior somatic cells that are in close contact with TFCs. While TFCs establish the number of niches, ICs appear to communicate with and regulate the number of GCs that can populate those niches. We hypothesize that TFCs and ICs do not regulate each other’s proliferation non-autonomously. However, we cannot test this hypothesis directly, as to our knowledge, no GAL4 drivers currently exist that are exclusively expressed in only TFCs or only ICs. Nevertheless, a number of lines of evidence support this hypothesis. First, reducing Hippo pathway activity in a subset of TFCs had no effect on IC number (S5H Fig.; S5 Table). Second, a double knockdown of *egfr* and *hpo* under the *tj*:*GAL4* driver reduced IC number but had no effect on TFC number (Fig. 5D). Third, loss of *germ cell-less* (*gcl*) function leads to reduced GC and IC numbers [49], but has no effect on ovariole number [81]. Given that ovariole number is largely determined by TFC number [50], it is likely that *gcl* ovaries have reduced ICs but not reduced TFCs. However, we note that both TFCs and ICs respond to hormonal cues provided by Ecdysone and Insulin signaling [6,8]. This suggests that growth of these somatic cell types may be accomplished through their response to systemic hormonal cues, rather than through non-autonomous effects of one somatic cell type on another.

While the Hippo pathway regulates proliferation of both ICs and TFCs, each cell type had a unique pattern of Hippo pathway activity during larval development, suggesting that the upstream regulatory cues of Hippo signaling are different for TFCs and ICs. In *Drosophila*, glial cells and wing disc cells activate the Hippo pathway using different combinations of upstream regulators [18], indicating that the Hippo pathway can interact with a unique set of upstream regulatory genes depending on the cell type. Addressing these cell type-specific differences in Hippo pathway activation in future studies will elucidate how the Hippo pathway is regulated locally during development of complex organs to establish organ size.

Another notable difference between Hippo pathway operation in ICs and TFCs is its differential interactions with the EGFR and JAK/STAT pathways in distinct ovarian cell types. In *Drosophila* intestinal stem cell development and stem cell-mediated regeneration [37,38,39,68], as well as in eye imaginal discs [64,67], the Hippo pathway regulates proliferation of these tissues via interactions with both the EGFR and JAK/STAT pathways. In contrast, the Hippo pathway acts in parallel with but independently of both pathways to regulate the maturation of *Drosophila* ovarian follicle cells [25,36]. We do not know what mechanisms determine whether the Hippo pathway interacts with EGFR signaling, JAK/STAT signaling, or both in a given cell or tissue type. One mechanism that may be relevant, however, is the differential activation of specific ligands. For example, in the *Drosophila* eye disc, Hippo signaling interacts genetically with EGFR activity induced by *vein*, but not by any of the other three *Drosophila* EGFR ligands [31]. Similarly, constitutively active human YAP can upregulate transcription of *vein*, but not the other three EGFR ligands, in *Drosophila* wing imaginal discs [31]. That fact that *spi* ^*RNAi*^ driven in the soma does not rescue the *hpo* ^*RNAi*^ overproliferation phenotype in the ovary may indicate that other ligands, such as *vein*, are required for this EGFR-Hippo signaling interaction, or that the relevant EGFR ligands are expressed by GCs rather than the soma. Our results suggest that the larval ovary could serve as a model to examine whether differential ligand use within a single organ could modulate Hippo pathway activity during development.

### Hippo signaling in germ cells of the larval ovary

Previous reports [35,36] suggested that the Hippo pathway components were dispensable for the proliferation of adult GSCs. In contrast, we observed that *yki* controls proliferation of the larval GCs, albeit independently of *hpo* and *wts*. These contrasting results are likely due to the fact that Sun *et al*. [35] sought to detect conspicuous germ cell tumors in response to reduced Hippo pathway activity, whereas we manually counted GCs and in this way detected significant changes in GC number in response to *yki* knockdown or overexpression. Although *hpo*, *wts*, *ex* or *hipk* RNAi (Fig. 4A) and *hpo* null clones (Fig. 4F) suggested that *yki* activity in GCs was independent of the canonical Hippo kinase cascade, overexpression of *hpo* in GCs did decrease GC number (Fig. 4A). Taken together, our data suggest that although sufficiently high levels of *hpo* are capable of restricting Yki activity in GCs, *hpo* does not regulate *yki* in GCs in wild type ovaries.

A growing body of evidence shows that *hpo*-independent mechanisms for regulating Yki are deployed in stem cells of multiple vertebrate and invertebrate tissues. For example, in mammalian epidermal stem cells, YAP is regulated in a Hpo-independent manner by an interaction between alpha-catenin and adaptor protein 14–43 [82]. Similarly, the C-terminal domain of YAP that contains the predicted *hpo*-dependent phosphorylation sites is dispensable for YAP-dependent tissue growth in postnatal epidermal stem cells in mice [83]. Other known Hpo-independent regulators of Yki include the phosphatase PTPN14 and the WW domain binding protein WBP2, which were identified in mammalian cancer cell lines [84,85]. The flatworm *Macrostomum ligano* displays a requirement for *hpo*, *sav*, *wts*, *mats* and *yki* in regulating stem cell number and proliferation, although it is unknown whether *yki* operates independently of the core kinase cascade in this system [86]. In contrast, however, in the flatworm *Schmidtea mediterranea*, while *yki* plays a role in regulating stem cell numbers, *hpo*, *wts* and *Mer* appear dispensable for stem cell proliferation [87]. We hypothesize that, as in many other stem cell systems, the *Drosophila* germ line may use Yki regulators that are not commonly used in the soma to regulate proliferation. Further investigation into the Yki interacting partners in GCs will be needed to understand how Yki may be regulated non-canonically in establishing stem cell populations.

### A novel role for Hippo signaling in germ line-soma homeostasis

**Fig 7:**
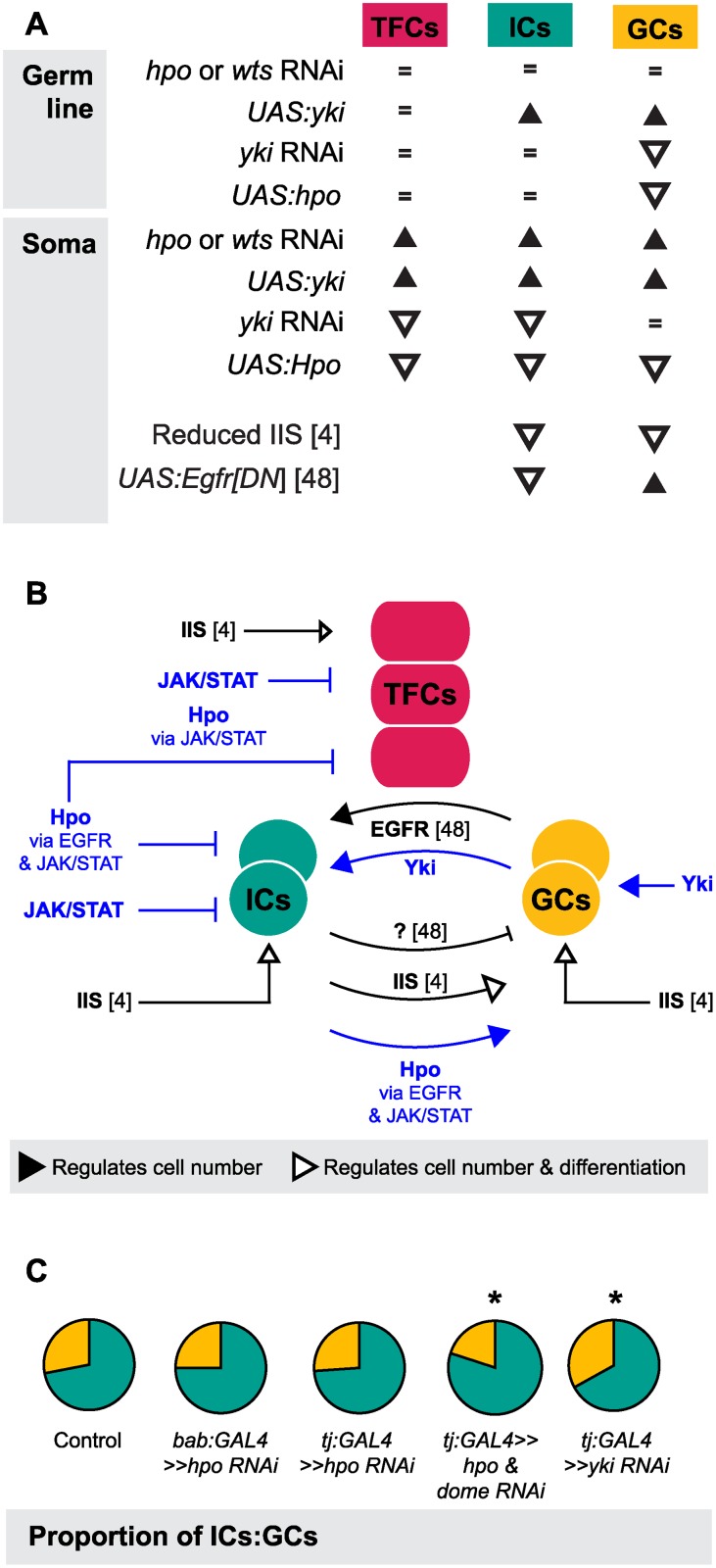
The Hippo pathway regulates coordinated growth of the soma and germ line. (A) Summary of changes in TFC, IC and GC numbers when expression of genes from various growth pathways were altered in our study and two other studies [6,49]. Black triangles indicate significant increase; white triangles indicate significant decrease; = indicate no significant change. (B) Model of how Hippo pathway influences coordinated proliferation of somatic cells and germ cells in the larval ovary. Contributions of the present study are indicated in blue; elements of the model derived from other studies [6,49] are indicated in black. The Hippo pathway interacts with JAK/STAT to regulate proliferation of TFCs, and interacts with EGFR and JAK/STAT pathways to regulate autonomous proliferation of ICs and non-autonomous proliferation of GCs. In addition, *yki* acts independently of *hpo* to influence proliferation of GCs in a non-canonical manner. (C) Summary of representative IC (green)/GC (yellow) proportions observed in our experiments, further elaborated in S7 Fig. Proportions of ICs and GCs are similar to controls when we knock down *hpo* or *wts* alone in the soma, but disrupting both *hpo* and EGFR or JAK/STAT pathway members leads to loss of proportional growth. Asterisk denotes *p*<0.05. See S6 Table for numerical values.

One of the most striking aspects of growth regulation in the larval ovary is the homeostatic growth of ICs and GCs during development. This homeostatic growth is critical to ensure establishment of an appropriate number of GSC niches that each contain the correct proportions of somatic and germ cells. We have summarized the available data on the molecular mechanisms that regulate the number of ICs and GCs (Fig. 7A) and our current understanding of how these mechanisms operate within and between the cell types that comprise the GSC niche (Fig. 7B). Previous work has shown that these mechanisms include the Insulin signaling and EGFR pathways. Insulin signaling function in the soma regulates differentiation and proliferation both autonomously in ICs and non-autonomously in GCs [6] (Fig. 7A, B). The EGFR pathway regulates homeostatic growth of both IC and GC numbers as follows: GCs produce the ligand Spitz that promotes survival of ICs, and ICs non-autonomously represses GC proliferation via an unknown regulator that is downstream of the EGFR pathway [49] (Fig. 7A, B). Our results add four critical new elements to the emerging model of soma-germ line homeostasis in the larval ovary (Fig. 7B, blue elements). First, *yki* positively and cell-autonomously regulates GC number independently of the canonical Hippo signaling pathway. Second, canonical Hippo signaling negatively and cell-autonomously regulates TFC number via JAK/STAT signaling, and IC number via both EGFR and JAK/STAT signaling. Third, JAK/STAT signaling also negatively regulates IC and TFC number in a cell-autonomous manner. Finally, Hippo signaling contributes to non-autonomous homeostatic growth of ICs and GCs in at least two ways: (1) Yki activity in GCs non-autonomously regulates IC proliferation; and (2) Hippo signaling activity in ICs non-autonomously regulates GC proliferation through the EGFR and JAK/STAT pathways. The latter relationship is, to our knowledge, the first report of a non-autonomous mechanism that ensures that GC number increases in response to increased IC number, without negatively affecting GSC niche differentiation or function.

Finally, we note that although IC number and GC number had been previously observed to affect each other non-autonomously [6,49], our experiments shed new light on the remarkable degree to which specific proportions of each cell type are maintained, and demonstrate the Hippo pathway’s involvement in this precise homeostasis. This proportionality was not maintained, however, in Hippo/ EGFR or Hippo/JAK/STAT pathway double knockdowns (Figs. 7, S7). This suggests that Hippo pathway-mediated proportional growth of ICs and GCs requires activity of not only the EGFR pathway, as previously reported [49], but also of the JAK/STAT pathway in the soma.

The proportional growth of these cell types maintained by the Hippo-EGFR-JAK/STAT pathway interactions we describe here suggests that the soma releases proliferation-promoting factors to the GCs, and that the GCs can process these signals to maintain optimal proportionality. Similarly, when GC number increased via *yki* overexpression in GCs, we noticed that IC number increased non-autonomously. Achieving specific numbers and proportions of distinct cell types within a single organ, and linking these processes to final organ size and function, are largely unexplained phenomena in developmental biology and organogenesis. By using the larval ovary as a system to address these problems, we have shown not only that the Hippo pathway is involved in these processes, but also that it can display remarkable complexity and modularity in regulating stem cell precursor proliferation and adjusting organ-specific stem cell niche number during development.

## Materials and Methods

### Fly stocks

Flies were reared at 25°C at 60% humidity with food containing yeast and in uncrowded conditions as previously described [50]. The following RNAi lines from the Bloomington Stock Center (B) [56] or the Vienna Drosophila RNAi Center (VDRC) [55] were used for knockdown: B33614 (*UAS*:*hpo* ^*RNAi*^), B34064 (*UAS*:*wts* ^*RNAi*^), B34067 (*UASyki* ^*RNAi*^), VDRC104523 (*UAS*:*yki* ^*RNAi*^), VDRC109281 (*UAS*:*ex* ^*RNAi*^), VDRC43267 (*UAS*:*egfr* ^*RNAi*^), VDRC19717 (*UAS*:*dome* ^*RNAi*^), B35363 (*UAS*:*hipk* ^*RNAi*^), B35481 (*UAS*:*sd* ^*RNAi*^). For overexpression of *hpo* or *yki* we used *w* ^*^ *; UAS*:*hpo/TM3 Sb* [19] and *w* ^*^ *; UAS*:*yki/TM6B* [21] (courtesy of D. Pan, Johns Hopkins University). GAL4 lines used were: *w; P{GawB}bab1* ^*Pgal4–2*^ */TM6*, *Tb* ^*1*^ (*bab*:*GAL4*, B6803), *P{UAS-Dcr-2*.*D}1*, *w* ^*1118*^ *; P{GAL4-nos*.*NGT}40* (*nos*:*GAL4*, B25751), *y w; P{w* ^*+mW*.*hs*^ *= GawB}NP1624* (*tj*:*GAL4*, Kyoto Stock Center, K104–055), *y w hs*:*FLP* ^*122*^ *; Sp/CyO; hh*:*GAL4/TM6B* (*hh*:*GAL4*, courtesy of L. Johnston, Columbia University), *w; P{w* ^*+mW*.*hs*^ *= GawB}ptc* ^*559*.*1*^ (*ptc*:*GAL4*, B2017). For GAL4 expression domain analysis, GAL4 lines were crossed to *w; P{w* ^*+mC*^ *= UAS-GFP*.*S65T}T2* (B1521). For clonal analysis of *hpo* and *yki* null alleles, the following lines were used: *w* ^*1118*^ *; P{ry* ^*+t7*.*2*^ *= neoFRT}*_*42D*_ *P{w* ^*+mC*^ *= Ubi GFP(S65T)nls}2R/CyO* (B5626), *w* ^*1118*^ *; P{ry* ^*+t7*.*2*^ *= neoFRT}*_*42D*_ *P{w* ^+t*^ *ry* ^+t*^ *= white-un1}*_*47A*_ (B1928), *P{ry* ^*+t7*.*2*^ *= hsFLP}1*, *w* ^*1118*^ *; Adv[* ^*1*^ *]/CyO* (B6), *hsFLP12 w* ^*^ *; P{ry* ^*+t7*.*2*^ *= neoFRT}42D yki* ^*B5*^ */CyO* [21] (courtesy of D. Pan, Johns Hopkins University), and *y* ^*^ *w* ^*^ *; P{ry* ^*+t7*.*2*^ *= neoFRT}*_*42D*_ *hpo* ^*BF33*^ */CyO (y+)* [57] (Courtesy of J. Jiang, University of Texas Southwestern Medical Center). For analysis of cell type numbers in flies homozygous for loss of function Hippo pathway alleles, we used *ex* ^*1*^ (B295; [54]) and y*w*eyFLP; FRT ^*42D*^ *yki* ^*DBO2*^ */ CyO* (Courtesy of K-L Guan, UCSD; [33]).

Validation of RNAi lines was provided by data from a number of independent experiments, as follows: (1) Immunohistochemistry against Hpo or Yki showed that RNAi against these genes reduced protein levels to levels indistinguishable from background in whole mounted larval ovaries (S1D, I Fig.). (2) Germ line clones of null alleles of *hpo* (*hpo* ^*BF33*^ [57]) or *yki* (*yki* ^*B5*^ [21]) had the same effect on germ cell number as RNAi against these genes driven in the germ line (Fig. 4F). (3) A null allele of *expanded* [54] had the same effect on TFC number, GC number and IC number as RNAi against Hippo pathway activity (S4 Fig., S2 Table). (4) Two different *yki* RNAi lines had the same effect on GC number (S3 Table). (5) Expression of pMAPK and Stat92E in the larval was reduced by RNAi against *egfr* or *spi* and *dome* or *upd1*, respectively (S8 Fig.). In addition, the *wts* ^*RNAi*^ and *dome* ^*RNAi*^ lines we used here have been independently validated by other studies [88,89].

### Immunohistochemistry

Larvae were all reared at 25°C at 60% humidity. Larval fat bodies were dissected in 1xPBS with 0.1% Triton-X, and fixed in 4% PFA in 1xPBS for 20 minutes at room temperature or overnight at 4°C. For tissues stained with the rat-Hippo antibody (courtesy of N. Tapon, London Research Institute), fat body tissue was fixed in freshly made PLP fixative [17] for 20 minutes. Tissues were stained as previously described [50]. Primary antibodies were used in the following concentrations: Mouse anti-Engrailed 4D9 (1:50, Developmental Studies Hybridoma Bank), guinea pig anti-Traffic Jam (1:3000–5000, courtesy of D. Godt, University of Toronto), rabbit anti-Vasa (1:500, courtesy of P. Lasko, McGill University), rabbit anti-Yorkie (1:400, courtesy of K. Irvine, Rutgers University), rat anti-Hippo (1:100, courtesy of N. Tapon, London Research Institute), chicken anti-Beta-galactosidase (1:200, Abcam), mouse anti-Alpha spectrin 3A9 (1:5, Developmental Studies Hybridoma Bank), rabbit anti-dpErk (1:300, Cell Signaling), rabbit anti-Stat92E (1:200, courtesy of E. Bach, New York University). We used goat anti-guinea pig Alexa 488, anti-mouse Alexa 488, Alexa 555, and Alexa 647, anti-rabbit Alexa 555, Alexa 647, anti-rat Alexa 568, and anti-chicken Alexa 568 at 1:500 as secondary antibodies (Life Technologies). All samples were stained with 10 mg/ml Hoechst 33342 (Sigma) at 1:500 to visualize nuclei, and some samples were stained with 0.1 mg/ml FITC-conjugated Phalloidin (Sigma) at 1:200 to visualize cell outlines. For GAL4 crosses, we crossed virgin females carrying the GAL4 construct with males carrying the UAS construct, and analyzed F1 LP stage larvae. Samples were imaged with Zeiss LSM 700, 710 or 780 confocal microscopes at the Harvard Center for Biological Imaging. Each sample was imaged in z-stacks of 1 μm thickness. For expression level analysis, laser settings were normalized to the secondary only control conducted in parallel to the experimental stain. Expression levels were quantified using Image J (NIH) and were normalized to nuclear stain intensity to control for staining level differences between samples.

### Cell type, ovariole number and egg-laying quantification

White immobile pupae were collected from uncrowded tubes (<100 larvae) for cell number analysis. All cell counts were obtained manually using Volocity (Perkin Elmer) after samples were randomized and coded to prevent bias; cells stained with Vasa were counted for germ cell number, and cells stained with Traffic Jam were counted for intermingled cell number. TF number and total TFC number were collected as described in [50]. Experimental crosses were compared to parental GAL4 and RNAi strains using a student’s t-test with unequal variance performed in Microsoft Excel. Changes in number were not considered significant unless *p* values were significant for both parental strains. For crosses where one or both parents were heterozygous for balanced GAL4 and/or UAS elements, sibling data from F1s carrying balancer chromosomes, rather than parental data, was collected as a control.

Adult ovariole number was counted in mated females that were 3–5 days post hatching from uncrowded vials kept in 25°C at 60% humidity. Adult ovaries were dissected in 1xPBS containing 0.1% Triton-X, and ovariole number was counted under a dissecting microscope by teasing apart ovariole strands using a tungsten needle. F1 ovariole number was compared to the ovariole number of siblings carrying balancer chromosomes for *bab*:*GAL4*, and to the *tj*:*GAL4* parental line for the *tj*:*GAL4* crosses.

Adult fecundity was measured by placing three females and one male in a vial for 24 hours, and counting total egg number per vial. Five replicates (vials) were performed for each treatment. The egg count was divided by the number of females to obtain the average egg number per female per 24 hours.

### Clonal analysis

P0 flies were mated (for *yki* ^*B5*^ clones: *w* ^*1118*^ *; P{ry* ^*+t7*.*2*^ *= neoFRT}*_*42D*_ *P{w* ^*+mC*^ *= Ubi GFP(S65T)nls}2R/CyO* x *hsFLP12 w* ^*^ *; P{ry* ^*+t7*.*2*^ *= neoFRT}42D yki* ^*B5*^ */CyO*; for *hpo* ^*BF33*^ clones: *P{ry* ^*+t7*.*2*^ *= hsFLP}1*, *w* ^*1118*^ *; P{ry* ^*+t7*.*2*^ *= neoFRT}*_*42D*_ *P{w* ^*+mC*^ *= Ubi GFP(S65T)nls}2R/CyO* x *y* ^*^ *w* ^*^ *; P{ry* ^*+t7*.*2*^ *= neoFRT}*_*42D*_ *hpo* ^*BF33*^ */CyO (y+)*; for control *w* clones: *P{ry* ^*+t7*.*2*^ *= hsFLP}1*, *w* ^*1118*^ *; P{ry* ^*+t7*.*2*^ *= neoFRT}*_*42D*_ *P{w* ^*+mC*^ *= Ubi GFP(S65T)nls}2R/CyO* x *w* ^*1118*^ *; P{ry* ^*+t7*.*2*^ *= neoFRT}*_*42D*_ *P{w* ^+t*^ *ry* ^+t*^ *= white-un1}47A*) and F1 eggs were collected for 8–12 hours at 25°C. L1 larvae were heat shocked at 37°C for 1 hour 36–48 hours after egg laying. Late L3 to LP stage ovaries were dissected, stained with 10 mg/ml Hoechst 4333 (Sigma) at 1:500, FITC-conjugated anti-GFP (1:500, Life Technologies), and rabbit anti-Vasa (1:500, courtesy of P. Lasko, McGill University), and imaged. GFP-negative mutant GC clone size (number of cells per clone) and GFP++ wild type twin spot clone size were counted manually.

## Supporting Information

S1 Fig Hippo pathway core components are expressed in the larval ovary. (A–C) Hippo protein is expressed ubiquitously in the larval ovary throughout development. (D) Hippo expression is strongly reduced in ovaries expressing RNAi against *hpo* under the somatic driver *tj*:*GAL4*, confirming specificity of the anti-Hpo antibody used in A–C and validating the RNAi line used. The decrease in Hpo protein levels observed throughout the ovary is likely due to the fact that the *tj*:*GAL4* driver is initially expressed in all somatic cells of the ovary, as previously reported [50,51]. (E) Secondary only control for Hippo antibody staining. Panels (B–E) were imaged at the same laser confocal settings. A–E show merged images with Hpo (A–D) or goat anti-Rat (E) in green, nuclear marker Hoechst 33342 in cyan, TFC marker anti-Engrailed in red (B–D), and IC marker anti-Traffic Jam in orange (B and C). B’-I’ show Hpo (A’–D’) or goat anti-Rat (E’) signal only. (F–H) Yorkie is detected in all somatic cells during larval ovarian development. (I) Yorkie expression is undetectable in ovaries expressing RNAi against *yki* using the somatic driver *bab*:*GAL4*, confirming specificity of the anti-Yki antibody used in F–H and validating the RNAi line used. The decrease in Yki protein levels observed throughout the ovary is likely due to the fact that the *bab*:*GAL4* driver is initially expressed in all somatic cells of the ovary, as previously reported [50,51]. (J) Secondary only control for Yki antibody. F–J show merged images with Yki (F–I) or goat anti-rabbit (J) in green, nuclear marker Hoechst 33342 in cyan, and TFC marker anti-Engrailed in red (G–I). F’–J’ show Yki (F’–I’) or goat anti-Rabbit (J’) signal only. Panels in (H–J) were taken at the same laser confocal settings. Green: Hippo or Yorkie; cyan: nuclei; red: Engrailed; orange: Traffic Jam. Scale bar = 10 μm.
(EPS)

S2 Fig Expression pattern of Hippo pathway activity reporter lines in larval ovarian cell types. Expression of (A–D, K) *diap1-LacZ* and (E–H, L) *bantam*-GFP reporters in larval ovarian cell types. (A) Engrailed-positive cells beginning to differentiate into disc-shaped TFCs express *diap1-LacZ*. (B) TFCs within a TF stack in mid-late L3 do not have strong *diap1* expression. (C–D) ICs and GCs express *diap1*. A–D show merged images with *diap1-lacZ* in green, nuclear marker Hoechst 33342 in cyan, TFC marker anti-Engrailed in red (A–B), and GC marker anti-Vasa in white (C–D). A’-D’ show *diap1-lacZ* signal only. (E–H) Expression of the *bantam-GFP* sensor line in larval ovarian cell types. The reporter line contains a GFP construct with three *bantam* miRNA target sites, so that GFP mRNA is degraded when *bantam* is expressed; GFP expression therefore indicates to little or no *bantam* expression. (E) Early TFCs express *bantam* (GFP expression is not detected). (F) TFCs in a mature TF express little to no detectable *bantam* (GFP expression is detected). (G) Low levels of GFP are detected in ICs, suggesting that *bantam* is expressed. (H) GCs express little or no detectable *bantam* (GFP expression is detected). Arrowheads point to an example of the specific cell types in each column. E–H show merged images with *bantam-*GFP sensor in green, nuclear marker Hoechst 33342 in cyan, and TFC marker anti-Engrailed in red (A). E’-H’ show *bantam-*GFP sensor signal only. Green: β-gal (A–B) or GFP (E–H); cyan: nuclei; red: Engrailed; white: Vasa (C–D). Scale bar = 10 μm. (I–L) Quantification of relative intensity of (I) Yki, (J) *expanded*-LacZ, (K) *diap1*-LacZ, and (L) the bantam-GFP sensor in early and mid L3 TFCs, ICs, and GCs. Error bars denote confidence intervals. n = 5 per measurement.
(EPS)

S3 Fig GFP expression driven by *traffic-jam* and *nanos* GAL4 during larval ovarian development. (A–D) *tj*:*GAL4* is expressed in most somatic cells in early larval development. Expression becomes confined to posterior cells in L3, persisting in a few TFCs and anterior patches of somatic cells. Expression in TFCs is strongest while TF stacking is occurring (arrowheads). GCs do not express *tj*:*GAL4*. (E) An anti-Traffic Jam antibody (green) detects a subset of the cells that express the *tj*:*GAL4* driver. (F–H) *nos*:*GAL4* is specific to GCs throughout larval ovarian development.. Green: GFP in A–D, F–I; Traffic Jam in E; blue: nuclei in all panels; red: Engrailed in all panels; orange: Traffic Jam in F; white: Vasa in A, E and I. Scale bar = 10 μm.
(EPS)

S4 Fig Homozygous mutants of Hippo pathway components significantly influence TFC, IC, and GC number. Percent difference of (A) TFCs, (B) ICs, and (C) GCs of *ex* ^*1*^ and *yki* ^*DBO2*^ homozygous mutants compared to *w* ^*1118*^ control line. + *p* = 0.06, * *p*<0.05, ** *p*<0.01. n = 10 for *ex* ^*1*^ and *w* ^*1118*^, and n = 6 for *yki* ^*DBO2*^. Numerical values can be found in S2 Table.
(EPS)

S5 Fig RNAi against Hippo pathway members driven by *ptc*:GAL4 or *hh*:GAL4 drivers does not significantly influence proliferation of larval ovarian cell types non-autonomously. (A–C) Expression domain of *ptc*:*GAL4* in L3 larval ovaries. (A) *ptc*:*GAL4* is expressed weakly in ICs and strongly in anterior somatic cells, but is not detected in TFCs (arrowheads in B and C). (D–F) Expression domain of *hh*:*GAL4* in L3 larval ovaries. *hh*:*GAL4* is expressed in a mosaic pattern in TFCs (arrowheads) with some expression in early and later stages of TF stacking, but not before TFC intercalation begins. (G–H) No significant difference in IC, TFC or TF number is observed when *hpo* ^*RNAi*^ or *wts* ^*RNAi*^ are expressed under (G) *ptc*:*GAL4* or (H) *hh*:*GAL4* drivers. Green bars: ICs; red bars: TFCs; pink bars: TFs.
(EPS)

S6 Fig Spectrosome morphology does not change when *yki* activity is altered in GCs. Alpha-spectrin staining (green) in LP stage GCs of (A) *nos*:*GAL4>>yki* ^*RNAi*^ and (C) *nos*:*GAL4>>UAS-yki* larvae and their siblings (controls: B and D). Round spectrosomes (green), indicating germ cells (red) that have not initiated oogenesis, are found in most GCs at this stage in all four genotypes. Scale bar = 10 μm.
(TIF)

S7 Fig ICs and GCs generally maintain homeostatic growth when Hippo pathway activity is reduced in the soma. Pie charts show proportion of ICs (green) and GCs (yellow) when we knocked down (A) Hippo pathway members alone, or in combination with (B) EGFR signaling pathway components or (C) JAK/STAT signaling pathway components using *bab*:*GAL4* and *tj*:*GAL4*. * denotes *p*<0.05, and ** denotes *p*<0.01. See S6 Table for numerical values.
(EPS)

S8 Fig RNAi against EGFR and JAK/STAT pathway components reduce respective pathway activity in the larval ovary. Relative intensity of (A) anti-pMAPK fluorescence in WT compared to *egfr* or *spi* RNAi expressed under *tj*:*GAL4* (n = 5), and (B) anti-Stat92E fluorescence in WT compared to *dome* or *upd1* RNAi expressed under *tj*:*GAL4* (n = 5) in L3 larval ovaries. + *p* = 0.06, * *p*<0.05.
(EPS)

S1 Table Summary of mean TFC and TF number in LP stage ovaries of genotypes used in RNAi analysis. Abbreviated names for GAL4 drivers are indicated in parentheses in leftmost column of first three rows. SD = standard deviation. Two-tailed t-tests were conducted for analysis and *p*-values are reported in columns compared to the UAS-RNAi parental strain (vs RNAi), GAL4 parental strain (vs GAL4), or the sibling (Sib) carrying balancers (vs Sib). Red shading indicates significant differences *p*≤0.01 (indicated by ** in Fig. 2); yellow shading indicates significant differences 0.01<*p*≤0.05 (indicated by * in Fig. 2); orange shading indicates near-significant differences 0.05<*p*≤0.1 (indicated by + in Fig. 2). VDRC indicates line 104523 from the Vienna Drosophila RNAi Center; TRiP indicates Transgenic RNAi Project line 34067 from the Bloomington Stock Center.
(PDF)

S2 Table Summary of mean ovariole, TFC, IC and GC number in ovaries of genotypes used in mutant analysis. SD = standard deviation. Two-tailed t-tests were conducted for analysis and *p*-values are reported in columns compared to the OregonR for ovariole number, and compared to *w* ^*1118*^ for TFC, IC and GC number. Red shading indicates significant differences *p*≤0.01; yellow shading indicates significant differences 0.01<*p*≤0.05; orange shading indicates near-significant differences 0.05<*p*≤0.1.
(PDF)

S3 Table Summary of mean IC and GC number in LP stage ovaries of genotypes used in RNAi analysis. Abbreviated names for GAL4 drivers are indicated in parentheses in leftmost column of first three rows. SD = standard deviation. Two-tailed t-tests were conducted for analysis and *p*-values are reported in columns compared to the UAS-RNAi parental strain (vs RNAi), GAL4 parental strain (vs GAL4), or the sibling (Sib) carrying balancers (vs Sibs). Red shading indicates significant differences *p*≤0.01 (indicated by ** in Figs. 3 and 4); yellow shading indicates significant differences 0.01<*p*≤0.05 (indicated by * in Fig. 3); orange shading indicates near-significant differences 0.05<*p*≤0.1 (indicated by + in Fig. 3). VDRC indicates line 104523 from the Vienna Drosophila RNAi Center; TRiP indicates Transgenic RNAi Project line 34067 from the Bloomington Stock Center.
(PDF)

S4 Table Summary of mean TFC, IC and GC number in LP stage ovaries of genotypes used in double RNAi analysis. SD = standard deviation. Two-tailed t-tests were conducted for analysis and *p*-values are reported in columns compared to the wild type control (vs WT) and the *hpo* RNAi control (vs *hpo* sib). Red shading indicates significant differences *p*≤0.01 (indicated by ** in Figs. 5 and 6); yellow shading indicates significant differences 0.01<*p*≤0.05 (indicated by * in Figs. 5 and 6); orange shading indicates near-significant differences 0.05<*p*≤0.1.
(PDF)

S5 Table Summary of TFC and IC number in *ptc*:*GAL4* and *hh*:*GAL4* analysis. SD = standard deviation. Two-tailed t-tests were conducted for analysis and *p*-values are reported in columns compared to the UAS-RNAi parental strain (vs RNAi) or the GAL4 parental strain (vs GAL4). Red shading indicates significant differences *p*≤0.01; yellow shading indicates significant differences 0.01<*p*≤0.05; orange shading indicates near-significant differences 0.05<*p*≤0.1.
(PDF)

S6 Table Summary of GC-IC proportion for single and double RNAi experiments influencing IC and/or GC number. Two-tailed t-tests were conducted for analysis and *p*-values are reported in columns compared to the RNAi parental control (vs RNAi), the GAL4 parental control (vs GAL4) or and the *hpo* RNAi control (vs *hpo* sib). Red shading indicates significant differences *p*≤0.01; yellow shading indicates significant differences 0.01<*p*≤0.05 (indicated by * in Figs. 7 and S6); orange shading indicates near-significant differences 0.05<*p*≤0.1.
(PDF)
