# Supplementary figures and images for: The Hippo Pathway Regulates Homeostatic Growth of Stem Cell Niche Precursors in the Drosophila Ovary
Source: PLoS Genet. 2015 Feb 2;11(2):e1004962. doi: 10.1371/journal.pgen.1004962 (PMC4333732; doi:10.1371/journal.pgen.1004962)

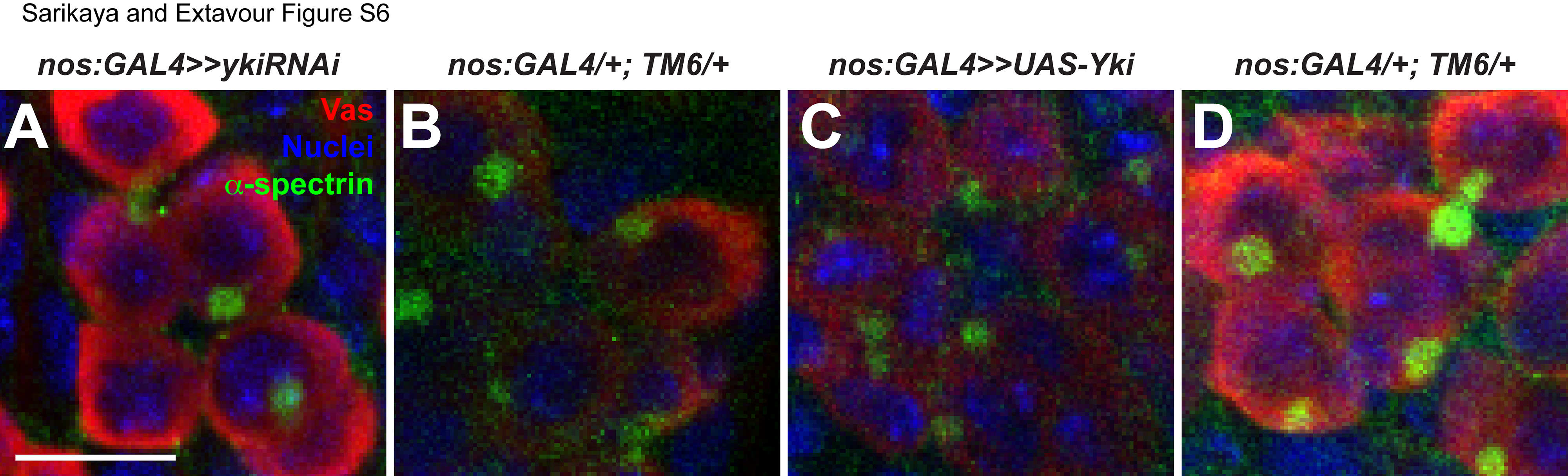

Supplement: S6 Fig — Alpha-spectrin staining (green) in LP stage GCs of (A) nos:GAL4>>yki RNAi and (C) nos:GAL4>>UAS-yki larvae and their siblings (controls: B and D). Round spectrosomes (green), indicating germ cells (red) that have not initiated oogenesis, are found in most GCs at this stage in all four genotypes. Scale bar = 10 μm. (TIF) [file pgen.1004962.s006.tif]
