# Supplementary material for: The Hippo Pathway Regulates Homeostatic Growth of Stem Cell Niche Precursors in the Drosophila Ovary
Source: PLoS Genet. 2015 Feb 2;11(2):e1004962. doi: 10.1371/journal.pgen.1004962 (PMC4333732; doi:10.1371/journal.pgen.1004962)
Supplement: S1 Table — Abbreviated names for GAL4 drivers are indicated in parentheses in leftmost column of first three rows. SD = standard deviation. Two-tailed t-tests were conducted for analysis and p-values are reported in columns compared to the UAS-RNAi parental strain (vs RNAi), GAL4 parental strain (vs GAL4), or the sibling (Sib) carrying balancers (vs Sib). Red shading indicates significant differences p≤0.01 (indicated by ** in Fig. 2); yellow shading indicates significant differences 0.01<p≤0.05 (indicated by * in Fig. 2); orange shading indicates near-significant differences 0.05<p≤0.1 (indicated by + in Fig. 2). VDRC indicates line 104523 from the Vienna Drosophila RNAi Center; TRiP indicates Transgenic RNAi Project line 34067 from the Bloomington Stock Center. (PDF) [file pgen.1004962.s009.pdf]

Supporting Table S1

	TFC Number					TF Number					
**Genotype**	**TFC#**	**SD**	**vs RNAi**	**vs GAL4**	**vs Sib**	**TF #**	**SD**	**vs RNAi**	**vs GAL4**	**vs Sib**	**n**
Controls											
bab:GAL4 (bab)	143.6	23.0				19.1	2.9				10
tj:GAL4 (tj)	148.0	16.8				19.5	2.6				10
nos:GAL4 (nos)	156.8	22.4				22.5	2.6				10
UAS-hpo^{RNAi}	169.9	13.5				22.2	1.9				10
UAS-wts^{RNAi}	157.1	15.1				20.9	2.0				10
UAS-yki^{RNAi}	153.5	14.8				21.3	2.2				10
Experimental											
bab>>hpo^{RNAi}	201.8	21.9	<0.01	<0.01		25.8	2.2	<0.01	<0.01		10
bab>>wts^{RNAi}	188.6	15.8	<0.01	<0.01		24.9	1.9	<0.01	<0.01		10
bab>>yki^{RNAi}	124.2	11.8	<0.01	0.02		18.3	1.3			0.01	10
tj>>hpo^{RNAi}	186.7	15.3	0.02	<0.01		25.5	2.0	<0.01	<0.01		10
tj>>wts^{RNAi}	188.3	31.6	0.01	<0.01		23.6	3.7	0.04	0.01		10
tj>>yki^{RNAi}	126.5	24.3	<0.01	0.03		17.1	3.0	<0.01	0.07		10
tj>>UAS-yki	183.0	28.2			0.04	26.0	4.4			<0.01	10
tj>>UAS-yki Sib	156.5	24.5				20.1	3.7				10
tj>>UAS-hpo	123.4	16.4			0.02	15.3	2.1			0.04	10
tj>>UAS-hpo Sib	144.3	20.2				17.5	2.2				10
nos>>hpo^{RNAi}	154.1	17.7	0.04	0.77		21.1	2.1	0.20	0.24		10
nos>>wts^{RNAi}	169.8	33.5	0.29	0.32		22.6	4.3	0.21	0.95		10
nos>>yki^{RNAi} (VDRC)	160.6	18.9	0.69	0.36		22.1	2.6	0.73	0.47		10
nos>>yki^{RNAi} (TRiP)	152.6	24.5			0.65	20.9	3.2			0.19	10
nos>>yki^{RNAi} *Sib*	156.5	18.9				22.3	2.6				10
nos>>UAS-yki	148.9	17.1			0.37	21.2	2.4			0.02	10
nos>>UAS-yki Sib	142.3	14.9				18.7	1.9				10
nos>>UAS-hpo	149.0	16.4			0.07	20.2	2.1			<0.01	10
nos>>UAS-hpo Sib	134.0	17.0				17.4	1.9				10
