# Supplementary material for: The Hippo Pathway Regulates Homeostatic Growth of Stem Cell Niche Precursors in the Drosophila Ovary
Source: PLoS Genet. 2015 Feb 2;11(2):e1004962. doi: 10.1371/journal.pgen.1004962 (PMC4333732; doi:10.1371/journal.pgen.1004962)
Supplement: S2 Table — SD = standard deviation. Two-tailed t-tests were conducted for analysis and p-values are reported in columns compared to the OregonR for ovariole number, and compared to w 1118 for TFC, IC and GC number. Red shading indicates significant differences p≤0.01; yellow shading indicates significant differences 0.01<p≤0.05; orange shading indicates near-significant differences 0.05<p≤0.1. (PDF) [file pgen.1004962.s010.pdf]

Supporting Table S2

	Ovariole Number			TFC Number			IC Number			GC Number			
Genotype	ON	SD	vs Control	TFC#	SD	vs Control	IC #	SD	vs Control	GC #	SD	vs Control	n
Control													
Oregon R	17.6	2.4											10
w[1118]				137.8	14.6		358.2	49.3		147.5	14		10
Mutants													
yki[DBO2]				155.1	19	0.06	515.5	75.8	<0.01	212.3	97.3	0.05	6
ex[1]	21.3	5.4	<0.01	185.4	18.9	<0.01	577.1	112	<0.01	200.8	56	<0.01	10
