# Supplementary material for: The Hippo Pathway Regulates Homeostatic Growth of Stem Cell Niche Precursors in the Drosophila Ovary
Source: PLoS Genet. 2015 Feb 2;11(2):e1004962. doi: 10.1371/journal.pgen.1004962 (PMC4333732; doi:10.1371/journal.pgen.1004962)
Supplement: S3 Table — Abbreviated names for GAL4 drivers are indicated in parentheses in leftmost column of first three rows. SD = standard deviation. Two-tailed t-tests were conducted for analysis and p-values are reported in columns compared to the UAS-RNAi parental strain (vs RNAi), GAL4 parental strain (vs GAL4), or the sibling (Sib) carrying balancers (vs Sibs). Red shading indicates significant differences p≤0.01 (indicated by ** in Figs. 3 and 4); yellow shading indicates significant differences 0.01<p≤0.05 (indicated by * in Fig. 3); orange shading indicates near-significant differences 0.05<p≤0.1 (indicated by + in Fig. 3). VDRC indicates line 104523 from the Vienna Drosophila RNAi Center; TRiP indicates Transgenic RNAi Project line 34067 from the Bloomington Stock Center. (PDF) [file pgen.1004962.s011.pdf]

Supporting Table S3

Genotype	IC Number: IC #	SD	vs RNAi	vs GAL4	vs Sib	GC Number: GC #	SD	vs RNAi	vs GAL4	vs Sib	n
Controls											
bab:GAL4 (bab)	528.8	63.2				223.4	59.0				10
tj:GAL4 (tj)	466.7	124.9				140.7	23.7				10
nos:GAL4 (nos)	526.7	120.8				167.2	32.8				10
UAS-hpo^{RNAi}	477.2	123.4				218.8	40.7				10
UAS-wts^{RNAi}	543.6	79.0				187.9	43.8				10
UAS-yki^{RNAi} (VDRC)	470.0	49.6				226.1	67.1				10
UAS-ex^{RNAi}	n/a					188.2	23.2				10
UAS-hipk^{RNAi}	n/a					228.3	26.7				10
UAS-sd^{RNAi}	n/a					216.4	46.2				9
Experimental											
bab x hpo^{RNAi}	625.2	157.5	0.03	0.09		298.4	44.3	<0.01	<0.01		10
bab x wts^{RNAi}	638.4	119.8	0.02	0.05		265.8	49.8	<0.01	0.09		10
bab x yki^{RNAi} (VDRC)	266.8	74.9			<0.01	211.5	38.4			0.76	10
bab x yki^{RNAi} *Sib*	462.7	41.0				207.5	17.7				10
tj x hpo^{RNAi}	818.2	124.5	<0.01	<0.01		267.2	24.3	<0.01	<0.01		10
tj x wts^{RNAi}	796.1	168.0	<0.01	<0.01		271.5	57.9	<0.01	<0.01		10
tj x yki^{RNAi} (VDRC)	322.5	83.6	<0.01	<0.01		157.7	43.2	<0.01	0.28		10
tj x UAS-yki	1186.9	382.7			<0.01	329.5	23.7			0.047	10
tj x UAS-yki Sib	477.7	111.2				275.1	77.4				10
tj x UAS-hpo	301.6	40.8			<0.01	166.6	35.3			0.04	10
tj x UAS-hpo Sib	505.7	125.7				219.4	65.3				10
nos x hpo^{RNAi}	609.7	95.5	0.01	0.11		193.9	39.4	0.18	0.11		10
nos x wts^{RNAi}	573.4	123.1	0.53	0.40		202.8	36.8	0.42	0.03		10
nos x yki^{RNAi} (VDRC)	477.6	86.1	0.81	0.31		136.5	33.3	<0.01	0.05		10
nos x yki^{RNAi} (TRiP)	424.0	108.0			0.47	120.5	15.0			<0.01	10
nos x UAS-yki	495.0	108.5			<0.01	315.7	97.7			<0.01	10
nos x UAS-yki Sib	353.6	86.5				167.0	34.9				10
nos x UAS-hpo	492.5	94.4			0.53	97.9	14.2			<0.01	10
nos x UAS-hpo Sib	517.6	81.1				206.5	31.0				10
Nos x UAS-YkiS168A						/239,1	91.6	n/a	0.03		8
nos x ex^{RNAi}	n/a					176.1	29.8	0.32	0.53		10
nos x hipk^{RNAi}	n/a					181.9	37.8	<0.01			10
nos x sd^{RNAi}	n/a					132.1	18.8	<0.01	<0.01		10
nos x hpo/wts/ex^{RNAi}	n/a					237.1	41.6				10
nos x hpo/wts/ex^{RNAi} *Sib*	n/a					239.2	33.0				9
