# Supplementary material for: The Hippo Pathway Regulates Homeostatic Growth of Stem Cell Niche Precursors in the Drosophila Ovary
Source: PLoS Genet. 2015 Feb 2;11(2):e1004962. doi: 10.1371/journal.pgen.1004962 (PMC4333732; doi:10.1371/journal.pgen.1004962)
Supplement: S4 Table — SD = standard deviation. Two-tailed t-tests were conducted for analysis and p-values are reported in columns compared to the wild type control (vs WT) and the hpo RNAi control (vs hpo sib). Red shading indicates significant differences p≤0.01 (indicated by ** in Figs. 5 and 6); yellow shading indicates significant differences 0.01<p≤0.05 (indicated by * in Figs. 5 and 6); orange shading indicates near-significant differences 0.05<p≤0.1. (PDF) [file pgen.1004962.s012.pdf]

Supporting Table S4

Genotype	TFC Number				IC Number				GC Number				
Genotype	TFC#	SD	vs WT	vs *hpo* sib	IC #	SD	vs WT	vs *hpo* sib	GC #	SD	vs WT	vs *hpo* sib	n
EGF pathway													
WT control: *spi*^{RNAi}/+; *hpo*^{RNAi}/+	127.9	16.9			432.4	100.3			164.5	23.2			10
tjGAL4/+;*hpo*^{RNAi}/+ [*egfr*^{RNAi} cross]	151.0	15.0	<0.01		709.8	144.6	<0.01		237.5	92.1	0.03		10
tjGAL4/*egfr*^{RNAi};*hpo*^{RNAi}/+	153.3	12.1	<0.01	0.72	577.7	87.8	<0.01	0.03	161.6	30.8	0.82	0.03	9
tjGAL4/*egfr*^{RNAi};+	131.9	11.4	0.54	<0.01	396.5	70.1	0.36	<0.01	205.8	55.8	0.04	0.36	10
tjGAL4/+;*hpo*^{RNAi}/+ [*spi*^{RNAi} cross]	165.4	21.8	<0.01		688.1	91.1	<0.01		245.0	32.7	<0.01		9
tjGAL4/*spi*^{RNAi};*hpo*^{RNAi}/+	171.3	16.9	<0.01	0.50	632.3	103.0	<0.01	0.24	185.6	79.6	0.43	0.05	9
tjGAL4/*spi*^{RNAi};+	137.2	9.2	0.14	<0.01	447.1	76.6	0.72	<0.01	185.2	29.9	0.10	<0.01	10
JAK/STAT pathway													
WT control: *upd1*^{RNAi}/+; *hpo*^{RNAi}/+	134.9	13.8			418.9	87.4			163.9	22.3			9
tjGAL4/+;*hpo*^{RNAi}/+ [*dome*^{RNAi} cross]	162.5	26.3	<0.01		680.1	110.4	<0.01		293.9	55.9	<0.01		9
tjGAL4/*dome*^{RNAi};*hpo*^{RNAi}/+	138.4	19.6	0.64	0.04	516.3	111.1	0.05	<0.01	154.7	36.6	0.52	<0.01	10
tjGAL4/*dome*^{RNAi};+	114.4	12.1	<0.01	<0.01	363.0	31.9	0.07	<0.01	157.6	30.9	0.85	<0.01	10
tjGAL4/+; *hpo*^{RNAi}/+ [*upd1*^{RNAi} cross]	155.2	24.4	<0.01		783.9	174.3	0.03		200.6	42.4	<0.01		9
tjGAL4/*upd1*^{RNAi}; *hpo*^{RNAi}/+	163.6	31.7	<0.01	0.51	613.1	33.2	<0.01	<0.01	146.8	32.1	0.20	<0.01	9
tjGAL4/*upd1*^{RNAi}; +	136.4	16.1	0.82	0.07	396.6	105.1	0.63	<0.01	171.2	23.9	0.51	0.09	9
