# Supplementary material for: The Hippo Pathway Regulates Homeostatic Growth of Stem Cell Niche Precursors in the Drosophila Ovary
Source: PLoS Genet. 2015 Feb 2;11(2):e1004962. doi: 10.1371/journal.pgen.1004962 (PMC4333732; doi:10.1371/journal.pgen.1004962)
Supplement: S5 Table — SD = standard deviation. Two-tailed t-tests were conducted for analysis and p-values are reported in columns compared to the UAS-RNAi parental strain (vs RNAi) or the GAL4 parental strain (vs GAL4). Red shading indicates significant differences p≤0.01; yellow shading indicates significant differences 0.01<p≤0.05; orange shading indicates near-significant differences 0.05<p≤0.1. (PDF) [file pgen.1004962.s013.pdf]

Supporting Table S5

Genotype	TFC Number: TFC#	SD	vs RNAi	vs GAL4	TF Number: TF #	SD	vs RNAi	vs GAL4	IC Number: IC #	SD	vs RNAi	vs GAL4	n
Controls													
ptc:GAL4	145.8	18.9			19.5	2.1			624.0	67.6			10
hh:GAL4	137.5	20.8			18.1	2.3			568.6	95.8			10
UAS-hpo^{RNAi}	169.9	13.5			22.2	1.9			477.2	123.4			10
UAS-wts^{RNAi}	158.7	17.1			20.7	1.9			543.6	79.0			10
Experimental													
ptc x hpo^{RNAi}	145	10.0	<0.01	0.90	18.6	1.1	<0.01	0.24	586.8	83.8	0.30	0.03	10
ptc x wts^{RNAi}	142.9	18.4	0.06	0.76	18.7	2.7	0.07	0.49	667.1	75.5	0.20	<0.01	10
hh x hpo^{RNAi}	166.2	19.3	0.01	0.63	21.9	3.1	0.01	0.80	577.5	122.7	0.08	0.87	10
hh x wts^{RNAi}	165.56	12.9	<0.01	0.37	20.7	1.9	0.02	1.00	486.1	123.7	0.17	0.23	10
