# Supplementary material for: The Hippo Pathway Regulates Homeostatic Growth of Stem Cell Niche Precursors in the Drosophila Ovary
Source: PLoS Genet. 2015 Feb 2;11(2):e1004962. doi: 10.1371/journal.pgen.1004962 (PMC4333732; doi:10.1371/journal.pgen.1004962)
Supplement: S6 Table — Two-tailed t-tests were conducted for analysis and p-values are reported in columns compared to the RNAi parental control (vs RNAi), the GAL4 parental control (vs GAL4) or and the hpo RNAi control (vs hpo sib). Red shading indicates significant differences p≤0.01; yellow shading indicates significant differences 0.01<p≤0.05 (indicated by * in Figs. 7 and S6); orange shading indicates near-significant differences 0.05<p≤0.1. (PDF) [file pgen.1004962.s014.pdf]

Supporting Table S6

Single RNAi Genotype	GC-IC Proportion GC	IC	vs RNAi	vs GAL4	vs Sib
Controls					
bab:GAL4 (bab)	29%	71%			
tj:GAL4 (tj)	23%	77%			
nos:GAL4 (nos)					
UAS-hpo^{RNAi}	30%	70%			
UAS-wts^{RNAi}	25%	75%			
UAS-yki^{RNAi} (VDRC)	30%	70%			
Experimental					
bab x hpo^{RNAi}	32%	68%	0.59	0.12	
bab x wts^{RNAi}	29%	70%	0.09	0.86	
bab x yki^{RNAi} (VDRC)	45%	55%			<0.01
bab x yki^{RNAi} *Sib*	31%	69%			
tj x hpo^{RNAi}	25%	75%	0.03	0.37	
tj x wts^{RNAi}	25%	75%	0.74	0.22	
tj x yki^{RNAi} (VDRC)	33%	67%	0.02	<0.01	
tj x UAS-yki	22%	78%			<0.01
tj x UAS-yki Sib	35%	65%			
tj x UAS-hpo	35%	65%			<0.01
tj x UAS-hpo Sib	30%	70%			

Double RNAi Genotype	GC-IC Proportion GC	IC	vs WT	vs hpo Sib
EGF pathway				
WT control: spi^{RNAi}*/+; hpo*^{RNAi}*/+*	27%	73%		
tjGAL4/+;hpo^{RNAi}*/+ [egfr cross]*	24%	76%	0.10	
tjGAL4/egfr^{RNAi}*;hpo*^{RNAi}*/+*	22%	78%	<0.01	0.18
tjGAL4/egfr^{RNAi}*;+*	33%	67%	<0.01	<0.01
tjGAL4/+;hpo^{RNAi}*/+ [spi cross]*	26%	74%	0.51	
tjGAL4/spi^{RNAi}*;hpo*^{RNAi}	20%	80%	<0.01	<0.01
tjGAL4/spi^{RNAi}*;+*	29%	71%	0.49	0.13
JAK/STAT pathway				
WT control: upd1^{RNAi}*/+; hpo*^{RNAi}*/+*	28%	72%		
tjGAL4/+;hpo^{RNAi}*/+ [dome cross]*	30%	70%	0.48	
tjGAL4/dome^{RNAi}*;hpo*^{RNAi}*/+*	23%	77%	<0.01	<0.01
tjGAL4/dome^{RNAi}*;+*	30%	70%	0.16	0.81
tjGAL4/+; hpo^{RNAi}*/+ [upd1 cross]*	21%	79%	<0.01	
tjGAL4/upd1^{RNAi}*; hpo*^{RNAi}*/+*	19%	81%	<0.01	0.12
tjGAL4/upd1^{RNAi}*; +*	30%	70%	0.32	<0.01
